# The kinase p38α functions in dendritic cells to regulate Th2-cell differentiation and allergic inflammation

**DOI:** 10.1038/s41423-022-00873-2

**Published:** 2022-05-12

**Authors:** Miaomiao Han, Jingyu Ma, Suidong Ouyang, Yanyan Wang, Tingting Zheng, Peishan Lu, Zihan Zheng, Weiheng Zhao, Hongjin Li, Yun Wu, Baohua Zhang, Ran Hu, Kinya Otsu, Xinguang Liu, Ying Wan, Huabin Li, Gonghua Huang

**Affiliations:** 1 ENT Institute and Department of Otorhinolaryngology, Eye & ENT Hospital, Fudan University, 200031 Shanghai, China; 2 Shanghai Institute of Immunology, Shanghai Jiao Tong University School of Medicine, 200025 Shanghai, China; 3 Guangdong Provincial Key Laboratory of Medical Molecular Diagnostics, Guangdong Medical University, 523808 Dongguan, China; 4 Biomedical Analysis Center, Army Medical University, 400038 Chongqing, China; 5 Department of Cardiovascular Medicine, Graduate School of Medicine, Osaka University, Osaka, 565-0871 Japan; 6 School of Cardiovascular Medicine and Sciences, King’s College London, London, SE59NU UK; 7 Present Address: Tongji Hospital, Tongji Medical College, Huazhong University of Science and Technology, 430030 Wuhan, China; 8 Present Address: Department of Dermatology, Yueyang Hospital of Integrated Traditional Chinese and Western Medicine, Shanghai University of Traditional Chinese Medicine, 200437 Shanghai, China; 9 Present Address: Department of Clinical Laboratory Medicine, Shanghai Tenth People’s Hospital of Tongji University, 200072 Shanghai, China; 10 Present Address: Basic Department of Cancer Center, Shanghai Tenth People’s Hospital of Tongji University, 200072 Shanghai, China

**Keywords:** Allergy, p38α, dendritic cell, Th2, Inflammation, Mucosal immunology

## Abstract

Dendritic cells (DCs) play a critical role in controlling T helper 2 (Th2) cell-dependent diseases, but the signaling mechanism that triggers this function is not fully understood. We showed that p38α activity in DCs was decreased upon HDM stimulation and dynamically regulated by both extrinsic signals and Th2-instructive cytokines. p38α-specific deletion in cDC1s but not in cDC2s or macrophages promoted Th2 responses under HDM stimulation. Further study showed that p38α in cDC1s regulated Th2-cell differentiation by modulating the MK2−c-FOS−IL-12 axis. Importantly, crosstalk between p38α-dependent DCs and Th2 cells occurred during the sensitization phase, not the effector phase, and was conserved between mice and humans. Our results identify p38α signaling as a central pathway in DCs that integrates allergic and parasitic instructive signals with Th2-instructive cytokines from the microenvironment to regulate Th2-cell differentiation and function, and this finding may offer a novel strategy for the treatment of allergic diseases and parasitic infection.

## Introduction

Upon antigen stimulation, naïve CD4^+^ T cells can differentiate into distinct effector T helper (Th) cell subsets, such as Th1, Th2, and Th17 cells as well as more recently described Th9 cells, to direct protective immunity against distinct pathogens or into regulatory T (Treg) cells to maintain immune homeostasis [1–4]. T-cell fate is largely determined by the nature of antigen-presenting cells (APCs) [5]. Dendritic cells (DCs) are the most important APCs and play a key role in linking innate and adaptive immunity [6]. DCs are activated by microbial stimuli through their pattern recognition receptors, which then program the DCs to direct distinct CD4^+^ T-cell fates [6, 7,]. Although much progress has been made in understanding the role of DCs in the initiation of Th1, Th17 and iTreg responses [8], how DCs direct Th2-cell differentiation and function is not fully understood [5, 9, 10,].

Th2 cells play pivotal roles in antihelminthic immunity and are essential in the pathogenesis of allergic diseases, such as atopic dermatitis and airway allergy [10]. Recent studies have shown that DCs are necessary and sufficient to orchestrate Th2 responses [11–14]. DCs are highly heterogeneous, and different tissues contain different DC subsets with distinct functions [15–17]. The DC classification system was previously based on ontogeny, function and phenotype, which allowed cells to be subdivided into type 1 conventional or classical DCs (cDC1s), type 2 conventional or classical DCs (cDC2s), and plasmacytoid DCs (pDCs) [16]. Based on unsupervised analysis of conventional flow cytometry and mass cytometry data, which combined lineage markers and certain surface markers expressed on cDCs, such as XCR1, CD172a and CD11b, mouse cDC1s and cDC2s can be generally defined as XCR1^hi^CD24^hi^CD26^hi^CD11c^hi^MHCII^hi^CD11b^lo^CD172a^lo^F4/80^lo^CD64^lo^Lin^lo^FSC^lo^SSC^lo^ and CD11b^hi^CD172a^hi^CD26^hi^CD11c^hi^MHCII^hi^XCR1^lo^F4/80^lo^CD64^lo^Lin^lo^FSC^lo^SSC^lo^ cells, respectively [18]. These three main subsets have been identified in multiple species, including mice and humans, with high similarity among species [18]. Although another DC subset, termed merocytic DCs, has been recently reported, whether it represents a true DC subset remains to be defined [19]. A recent study using single-cell RNA-sequencing (scRNA-seq) analyses of DCs revealed more heterogeneity than previous classifications [20], which led to the identification of six main DC groups in human blood [21]. scRNA-seq analysis has also identified novel subsets and major cDC2 subsets that differentially express T-bet and RORγt in mice [22]. Moreover, earlier studies have long suggested that cDCs and pDCs are derived from common DC progenitors in the bone marrow [23]. However, scRNA-seq studies have also identified that mouse pDCs mostly develop from Ly6D^+^ lymphoid progenitors distinct from the myeloid lineage [20, 24,]. Notably, Langerhans cells were recently reclassified as macrophages rather than DCs [25]. Under inflammatory conditions, monocytes can be further differentiated into classical Ly6C^hi^ monocyte-derived DCs (mo-DCs) [26, 27,], and mouse and human mo-DCs also have a gene expression profile distinct from that of cDCs [16, 28,]. To study DC function in vivo, genetic tools based on the differential transcription factor dependence of mouse DC subset development are widely used, such as *Zbtb46* for cDCs, *Irf8* and *Batf3* for cDC1s, and *Irf4* for cDC2s [29–31]. Although extensive heterogeneity among DCs in homeostatic and pathological states has been observed through scRNA-seq, the roles of DC subsets in Th2 responses remain unclear, as contradictory conclusions have been made due to the use of different triggers, genetic and disease models, or experimental approaches in previous studies [32–46]. cDC2s are widely reported to dominantly orchestrate Th2-cell differentiation and Th2-mediated allergic disease [39, 40, 42, 45, 47, 48,], but cDC1s have also been found to be essential for Th2 responses [32, 38, 43, 44,]. As such, the detailed signaling pathways underlying the distinct functions of certain DC subsets involved in instructing Th2-cell differentiation remain to be established.

DCs instruct naïve CD4^+^ T-cell differentiation through antigen presentation, costimulatory molecules and polarizing cytokines [49]. Upon receipt of various stimulations, DCs express multiple Th2-polarized costimulatory molecules and cytokines, such as CD80 [50], CD86 [51], OX40L [52, 53,], IL-6 [54], IL-10 [55], TSLP [56], IL-33 [57] and the Notch ligands Jagged-1 and Jagged-2 [58]. DCs also secrete cytokines such as IL-12 to suppress Th2-cell differentiation [43, 44,]. Recent studies have also identified several transcription factors, such as STAT5, IRF4 and KLF4, involved in Th2 polarization [45, 46, 48, 59,]. However, how intracellular signaling in DCs orchestrates Th2 polarization is still not well understood. Moreover, the Th2-sentinel function of DCs requires not only the direct effects of allergens but also indirect activation through mucosal epithelial cells (ECs) or other innate immune cells [33]. Upon allergen stimulation, ECs secrete large amounts of Th2-instructive cytokines, such as IL-4, TSLP, IL-25, IL-33, and GM-CSF, which further program DCs to shape Th2-cell differentiation [33]. However, how these Th2-instructive cytokines act on DCs to direct Th2 responses is largely unknown. The p38 MAPK signaling pathway is one of the key pathways involved in responding to extracellular signals to regulate inflammatory responses [60]. Our previous studies have demonstrated that under various conditions, DCs can employ p38α signaling to regulate Th1-cell, Th17-cell, Tr1-cell, iTreg-cell and γδ T-cell differentiation and function [61–64]. However, how DCs use the p38α signaling pathway to regulate Th2-cell differentiation and Th2-mediated diseases has never been studied. Although the role of p38 in asthma pathogenesis has been established, most studies are based on the usage of p38 inhibitors or inducible deletion of p38α [65–67]. By using a combination of experimental approaches and multiple genetic mouse models, herein, we report that p38α signaling is important in DC-mediated regulation of Th2-cell differentiation and Th2-mediated allergic inflammation both in the skin and in the lungs, as well as in antiparasite immunity. p38α acted on cDC1s to regulate Th2-cell differentiation by modulating the MK2−c-FOS−IL-12 axis. Moreover, p38α mediated the crosstalk between DCs and Th2 cells during the sensitization phase, not the effector phase. We also found that this activity of p38α was conserved between mouse and human DCs and was regulated by both extrinsic signals and Th2-instructive cytokines. Thus, our study links allergic instructive signals and DC p38α-dependent IL-12 expression with Th2 responses and provides cellular and molecular mechanisms for the p38α-mediated regulation of susceptibility to allergic inflammation and antiparasite immunity.

## Materials and methods

### Mice

CD11c-Cre and *Mapk14*^fl/fl^ mice were generated as previously described [61, 68, 69,]. *Irf8*^fl/fl^, *Irf4*^fl/fl^, OT-II and zDC (*Zbtb46*)-Cre mice (Jac:028538) were obtained from The Jackson Laboratory. *Xcr*1-Cre mice were obtained from the Shanghai Model Organisms Center, Inc. LysM-Cre mice were kindly provided by Dr. Jinke Cheng (Shanghai Jiao Tong University School of Medicine, China). We used an *Irf8*-deficient mouse line (*Irf8*^fl/fl^CD11c-Cre mice) and an *Irf4*-deficient mouse line (*Irf4*^fl/fl^CD11c-Cre mice) to specifically target CD11c-expressing cDC1s and cDC2s in vivo, respectively [70]. By crossing *Mapk14*^fl/fl^ mice with *Irf8*^fl/fl^CD11c-cre mice or *Irf4*^fl/fl^CD11c-cre mice, we explored the role of p38α in cDC1s and cDC2s in vivo. To more specifically study the role of p38α in cDC1s, we used a recently developed *Xcr1-*Cre mouse line and crossed this mouse line with *Mapk14*^fl/fl^ mice to obtain mice with p38α-specific deletion in cDC1s [71]. We applied the LysM-Cre mouse line to specifically delete genes in mature macrophages or monocyte-derived cells. By crossing this mouse line with *Mapk14*^fl/fl^ mice, we obtained mice with p38α deletion in macrophages. CD45.1 and Rosa26-Cre-ER^T2^ mice were kindly provided by Prof. Bing Su (Shanghai Jiao Tong University School of Medicine, China). All mice used in this study had been backcrossed onto the C57BL/6 background for at least eight generations and were used at 6−10 weeks old unless otherwise noted. All mice were housed under specific pathogen-free conditions in the Animal Resource Center at the Shanghai Jiao Tong University School of Medicine. All animal experimental procedures were approved by the Institutional Animal Care and Use Committee of the Shanghai Jiao Tong University School of Medicine (A-2019-033).

### House dust mite (HDM)-induced allergic airway disease

Allergic airway disease was induced by HDM extract (RMB84M, Mite Dust, *Dermatophagoides pteronyssinus*; Endotoxin: 2144 EU/g, Greer Labs) as described previously with minor modification [72]. Briefly, 6- to 10-week-old mice were *intranasally* (*i.n*.) sensitized with 50 μg HDM on Days 0–2 and challenged with 10 µg HDM on Days 14–16 (in 40 µl PBS). The mice were sacrificed for analysis on Day 17. For the sensitization phase, 6- to10-week-old mice were *i.n*. sensitized with 50 μg HDM on Days 0–2 and sacrificed for analysis on Day 9. For IL-12 treatment, mice were given an administration of 50 ng recombinant murine IL-12p70 (PeproTech) *i.n*. during HDM administration.

### Airway hyperresponsiveness (AHR) detection

AHR w in response to increasing doses of aerosolized methacholine (Sigma–Aldrich) as measured 24 h after the last HDM challenge by a Buxco invasive measurement system (Buxco, USA) as described previously [73]. Briefly, mice were anesthetized and ventilated mechanically at a rate of 200 breaths per min and a tidal volume of 200 μl. Aerosolized PBS and increasing doses of methacholine (6.25, 12.5 and 25 mg/ml) were administered after the baseline data were established. Lung resistance is expressed as the mean RL = cm H_2_O ml^−1^ s^−1^.

### BALF collection and analyses

The BALF was harvested by intubating the mouse trachea and lavaging the lungs four times with 1 ml PBS. The first flush of BALF was collected for ELISA, while the cells in the four flushes were harvested for cell counts and flow cytometry after centrifugation.

### Lung histology

After BALF collection, the left lobes of lung explants were taken for staining with H&E or PAS as described previously [74]. Briefly, lung explants were fixed in 4% paraformaldehyde (PFA) and embedded in paraffin, and 5 µm sections were stained with H&E or PAS. Histological images were acquired with an Axio Vert.A1 microscope (ZEISS). The degree of lung inflammation in the H&E-stained sections was blindly quantified according to previously published criteria [75]. The quantification of mucus expression in the airway was performed as previously described [76].

### Lung mononuclear cell isolation

Lung mononuclear cells were prepared as previously described [77]. Briefly, lung tissues were sliced into small pieces and incubated at 37 °C for 45 min with collagenase IV (1 mg/ml; Life Technologies) in RPMI-1640 medium (HyClone) supplemented with 5% fetal bovine serum (FBS; HyClone), and cells were isolated by gradient centrifugation over 38% Percoll (GE Healthcare Life Sciences). After erythrocyte lysis with ACK lysis buffer (Gibco), the cells were harvested for analyses.

### Papain immunization

Six- to 10-week-old WT and p38α^ΔDC^ mice were *i.n*. immunized with 30 μg papain (Merck Millipore) on Days 0–4 and sacrificed for analysis on Day 5.

### Flow cytometry

For surface staining, cells were stained in PBS containing 1% (vol/vol) FBS with anti-ms (mouse) CD4 (RM4-5), anti-ms TCRβ (H57-597), anti-ms MHCII (M5/114.15.2), anti-ms CD11c (N418), anti-ms CD11b (M1/70), anti-ms Siglec F (E50-2440), anti-ms Ly6G (RB6-8C5), anti-ms CD40 (1C10), anti-ms CD80 (16-10A1), anti-ms CD86 (GL1), anti-ms CD103 (2E7), anti-ms CD90.1 (HIS51), anti-ms CD90.2 (53-2.1), anti-ms CD45.1 (A20), anti-ms F4/80 (BM8), anti-ms CD24 (M1/69), anti-ms CD19 (1D3), and anti-ms CD317 (PDCA-1, eBio927) antibodies; a Lineage-negative gating cocktail identifying the markers ms CD3 (17A2), CD5 (53-7.3), FcεR1 (MAR-1), NK1.1 (PK136), CD11b (M1/70), B220 (RA3-6B2) and CD11c (N418); anti-hu (human) Hematopoietic Lineage Cocktail; and 7-AAD. All the antibodies were purchased from eBioscience. Anti-hu HLA-DR (G46-6) and anti-hu CD11c (B-ly6) were purchased from BD Biosciences. For intracellular staining (ICS), cells were stimulated with PMA (Sigma–Aldrich) and ionomycin (Sigma–Aldrich) in the presence of GolgiStop (BD Biosciences) for 5 h and then stained according to the manufacturer’s instructions (BD Biosciences) with antibodies against ms IL-4 (11B11; eBioscience), ms IL-5 (TRFK5; BD Biosciences),ms IL-13 (eBio13A; eBioscience), ms IFNγ (XMG1.2; eBioscience), and ms IL-17 (eBio17B7; eBioscience). For transcription factor detection, cells were stained with the Foxp3/Transcription Factor Staining Buffer Set (eBioscience), anti-ms GATA3, anti-ms T-bet and anti-ms RORγt. For IL-12 detection, lung cells were stimulated with HDM for 8 h, with GolgiStop (BD Biosciences) added for the last 4 h, and then stained according to the ICS manufacturer’s instructions (BD Biosciences) with anti-ms IL-12p40 (C17.8; eBioscience), anti-ms IL-12p35 (4D10p35; eBioscience). For intracellular phosphorylation assays, cells were stained with anti-phospho-p38 (28B10; Cell Signaling Technology) according to the manufacturer’s instructions (BD Biosciences). T-cell proliferation was detected by anti-Ki67 (SolA15; eBioscience) staining according to the manufacturer’s instructions (eBioscience) or labeling with carboxyfluorescein diacetate succinimidyl diester (CFSE; Invitrogen). For cell apoptosis analysis, cells were stained with an Active Caspase-3 Apoptosis Kit (BD Biosciences) or CaspACE™ FITC-VAD-FMK In Situ Marker (Promega). Flow cytometry data were acquired on a BD FACSCanto^TM^ II or BD LSRFortessa^TM^ X-20 and were analyzed with FlowJo software (TreeStar).

### Fluorescein isothiocyanate (FITC)-induced contact hypersensitivity (CHS)

CHS was induced using the hapten FITC (Sigma–Aldrich) as described previously with minor modification [59]. In brief, 100 μl 0.5% FITC (Sigma–Aldrich) resuspended in acetone and DBP (Sigma–Aldrich) (at a ratio of 1:1) was painted onto the shaved skin of WT and p38α^ΔDC^ mice on Days 0 and 1. On Day 6, the baseline ear thickness was measured, and the mice were challenged by applying 20 μl of 0.5% FITC or vehicle onto the contralateral ear. Ear thickness was measured, and the mice were sacrificed for analysis 24 h later. Skin cells were prepared as previously described [63].

### Skin histology

Pieces of mouse ears were fixed in 4% PFA and embedded in paraffin, and 6 µm sections were stained with H&E and analyzed by microscopy. Inflammatory responses were scored as reported previously [78].

### Parasite egg immunization

A total of 5000 *Schistosoma japonicum* eggs in 50 µl PBS were injected *s.c*. into the hind footpad of WT and p38α^ΔDC^ mice. The draining popliteal LNs (dLNs) were harvested 7 days after immunization to perform an ex vivo SEA restimulation assay [43].

### RNA sequencing

WT and p38α^ΔDC^ mice were treated *i.n*. with 50 μg HDM, and 24 h later CD19^−^Siglec F^−^CD11c^+^MHCII^+^CD103^+^CD11b^−^ (cDC1s) and CD19^−^Siglec F^−^CD11c^+^MHCII^+^ CD103^−^CD11b^+^ (cDC2s) cells were sorted by FACS for RNA-sequencing analysis. Low-input RNA-seq libraries were constructed and analyzed according to a previously described method [79]. Genes detected in over 75% of the samples at >1 FPKM were retained for downstream analysis. Gene set enrichment analysis (GSEA) was performed using gene lists from the Molecular Signatures database, namely, the Molecular Signatures Database (MSigDB) hallmark gene set collection [80].

### Bone marrow-derived dendritic cell (BMDC) culture

BMDCs were cultured as previously described with minor modification [59]. Bone marrow cells collected from femurs and tibias were cultured in RPMI-1640 medium (HyClone) supplemented with 10% FBS (Gibco), 50 μM 2-mercaptoethanol (Sigma–Aldrich), penicillin and streptomycin (Invitrogen) in the presence of 10 ng/ml mouse granulocyte-macrophage colony-stimulating factor (mGM-CSF; R&D) and 10 ng/ml mIL-4 (R&D). The culture medium was replenished on Day 3, and the cells were harvested on Day 7. Bone marrow cells were cultured in the presence of 150 ng/ml mFlt3L (R&D) and harvested on Days 8−9 to collect Flt3L-DCs. For p38α deletion in Flt3L-DCs derived from p38α^CreER^ mice, 0.5 μM (Z)-4-hydroxytamoxifen (4-OHT; Sigma–Aldrich) was added on Day 4. The purity of both CD11c^+^ BMDC populations was >80%. Flt3L-cDC1s (CD11c^+^ B220^−^CD24^+^CD11b^−^) were sorted by FACS for coculture.

### Adoptive transfer experiment

For the mediastinal lymph node (mLN) cell-derived adoptive transfer experiment, 10 × 10^6^ mLN cells were isolated from CD45.1 mice 5 days after treatment with HDM and *i.n*. injected into WT and p38α^ΔDC^ recipient mice. The recipients were then *i.n*. challenged with 10 μg HDM for 3 consecutive days and analyzed 24 h after the last challenge. HDM-pulsed BMDC transfer was performed as previously described with minor modification [54]. In brief, WT mice were sensitized with HDM for 3 consecutive days (Days 0−2). On Day 14, 2 × 10^6^ WT or p38-deficient BMDCs pulsed with 50 μg HDM for 12 h were washed twice with PBS and *i.n*. transferred into HDM-sensitized WT recipients; analysis was performed 72 h after BMDC transfer.

### Protein analyses

The concentrations of IL-4, IL-5, IL-13, IL-17, and IFNγ in the BALF were measured by ELISA according to the manufacturer’s instructions (R&D; eBioscience). The HDM-specific IgE titer was determined by ELISA as previously described with minor modification [81]. Briefly, a 96-well plate (Corning™ Costar™) was coated with 10 μg/ml HDM overnight at 4 °C. The plate was washed and blocked for 1 h at 37 °C with assay diluent. After washing, serum samples were added and incubated for 2 h at room temperature. After another wash, biotin-conjugated anti-mouse IgE (eBioscience) was added and incubated for 1 h at room temperature, followed by the addition of streptavidin-HRP. Substrate Solution TMB (eBioscience) was added to each well. The plate was incubated at room temperature for 30 min, and 100 µl Stop Solution (2 N H_2_SO_4_) was added to each well. The OD values were read at 450 nm on a MultiSKAN GO microplate reader (Thermo). The HDM-specific IgG1 titer was determined by ELISA as previously described with minor modification [82]. Briefly, a 96-well plate (Corning™ Costar™) was coated with 5 µg/ml HDM overnight at 4 °C. The plate was washed and blocked for 1 h with assay diluent. Samples were added the next day and incubated for 3 h at room temperature after another wash. Biotin-conjugated anti-mouse IgG1 (eBioscience) was added and incubated for 1 h at room temperature, followed by the addition of streptavidin-HRP. Substrate Solution TMB (eBioscience) was added to each well. The plate was incubated at room temperature for 30 min, and 100 µl Stop Solution (2 N H_2_SO_4_) was added to each well. The OD values were read at 450 nm on a MultiSKAN GO microplate reader (Thermo). Immunoblot analysis was performed as described [61] with antibodies against p38, MK2 phosphorylated at Thr334, c-FOS phosphorylated at Ser32, β-actin (all from Cell Signaling Technology) and GAPDH (Proteintech).

### RNA analyses

Total RNA was isolated from lung tissues with TRIzol reagent (Invitrogen) and from cells with an RNeasy mini Kit (QIAGEN). Reverse transcription was performed with PrimeScript RT Master Mix (TAKARA) according to the manufacturer’s instructions. Quantitative PCR (qPCR) was carried out with SYBR Green PCR Master Mix (Applied Biosystems) on a Vii7 Real-Time PCR system (Applied Biosystems). The mRNA expression of mouse genes was normalized to that of *Hprt*, and that of human genes was normalized to that of *GAPDH*. The primers used were obtained from Primerbank: *Hprt*, forward primer: TCAGTCAACGGGGGACATAAA, reverse primer: GGGGCTGTACTGCTT AACCAG; *Il4*, forward primer: GGTCTCAACCCCCAGCTAGT, reverse primer: GCCGATGATCTCTCTCAAGTGAT; *Il17a*, forward primer: TCAGCGTGTCCAAACACTGAG, reverse primer: CGCCAAGGGAGTTAAAGACTT; *Ifng*, forward primer: GCCACGGCACAGTCATTGA, reverse primer: TGCTGATGGCCTGATTGTCTT; *Tbx21*, forward primer: AGCAAGGACGGCGAATGTT, reverse primer: GGGTGGACATATAAGCGGTTC; *Il10*, forward primer: CTTACTGACTGGCATGAGGATCA, reverse primer: GCAGCTCTAGGAGCATGTGG; *Mapk14*, forward primer: GAGGTGCCCGAACGATAC, reverse primer: TGGCGTGAATGATGGACT; *Il12p40*, forward primer: GTCCTCAGAAGCTAACCATCTCC, reverse primer: CCAGAGCCTATGACTCCATGTC; and human *IL5*, forward primer: AAGAGACCTTGGCACTGCTTTC, reverse primer: GGAACAGGAATCCTCAGAGTCT. Other primers, such as those for *Il5*, *Il13*, *Il33*, *Il25*, *Tslp* [83], *Gata3* [84], *Il9* [85], human *IL4*, *human IL13* [86], human *IL17* [87], human *IFNG*, human *GAPDH* [88], and human *IL12P40* [89], were used as described previously.

### Cell stimulation and culture

To evaluate ex vivo recall responses, mLN cells from HDM-sensitized mice were stimulated with 50 μg/ml HDM or PBS for 72 h for cytokine assays. For drug inhibitor treatment, cells were incubated with vehicle (DMSO), an MK2 inhibitor (Merck Calbiochem, 20 μM), or the AP1 inhibitor SR11302 (MedChemExpress, 10 μM) for 0.5−1 h before stimulation with HDM. For agonist stimulation, cells were stimulated with HDM in the presence or absence of the AP1 agonist recombinant mouse epidermal growth factor (EGF, Novoprotein). For mouse DC−T-cell coculture, flow cytometry-sorted lung DCs (CD11c^+^MHCII^high^), cDC1s, or cDC2s (purity > 95%) isolated from WT or p38α^ΔDC^ mice 24 h after HDM treatment and naïve OT-II CD4^+^ T cells (CD4^+^CD25^−^CD44^−^CD62L^+^, purity >99%) (1:10) were mixed in the presence of the OVA_323-339_ peptide, and then cells were harvested (48−72 h) for mRNA analysis, or the supernatant was harvested for ELISA. For cytokine treatment, cultures were supplemented with 0.5 ng/ml recombinant mouse IL-12 (BD Pharmingen), 10 ng/ml recombinant mouse TSLP (eBioscience) or 10 ng/ml recombinant mouse IL-33 (BioLegend). For drug inhibitor-treated DC−T-cell coculture, DCs were incubated with vehicle (DMSO) or a drug inhibitor in the presence of HDM for 5 h, harvested, washed twice with PBS, and then cocultured with naïve OT-II CD4^+^ T cells.

### Human DC and T-cell coculture

Human mo-DCs were prepared as described previously [90]. Briefly, peripheral blood mononuclear cells (PBMCs) were obtained from healthy donor blood by Ficoll-Hypaque (GE Healthcare) density centrifugation. CD14^+^ cells (purity > 95%) were isolated using the EasySep™ Human CD14 Positive Selection Kit (STEMCELL) and cultured in RPMI-1640 medium supplemented with penicillin/streptomycin, 10% FBS, recombinant hGM-CSF (100 ng/ml) and hIL-4 (100 ng/ml; both from R&D) for 6 days. Human mo-DCs were treated with 10 µM SB203580 and HDM for 24 h, washed extensively and cocultured with human blood naïve CD4^+^ T cells isolated using the Naïve CD4^+^ T Cell Isolation Kit II (Miltenyi Biotec) at a ratio of 1:10. After 7 days of coculture, live T cells were purified and stimulated with plate-bound anti-human CD3 (UCHT1; BioLegend) for 5 h and then harvested for mRNA analysis. PBMCs were collected from allergic rhinitis (AR) patients and stimulated with the p38 inhibitor SB203580 or DMSO (vehicle) for 8 h, with GolgiStop added to the culture medium for the last 5 h. Cells were harvested, and IL-12p40 (C8.6, eBioscience) expression in DCs was detected by ICS. This study was approved by the Ethics Committee of the Eye & ENT Hospital of Fudan University (2017-0301). Informed consent was obtained from all volunteers.

### Statistical analysis

Statistical analysis was performed using Prism 5.0 or Prism 8.0 (GraphPad). Results are represented as the mean ± SEM. Statistical significance was determined by an unpaired Student’s *t* test or two-way ANOVA with Bonferroni’s post-test, as indicated in the figure legends (*, *P* < 0.05; **, *P* < 0.01).

## Results

### DC-specific p38α deletion renders mice susceptible to HDM-induced allergic asthma

**Fig. 1:**
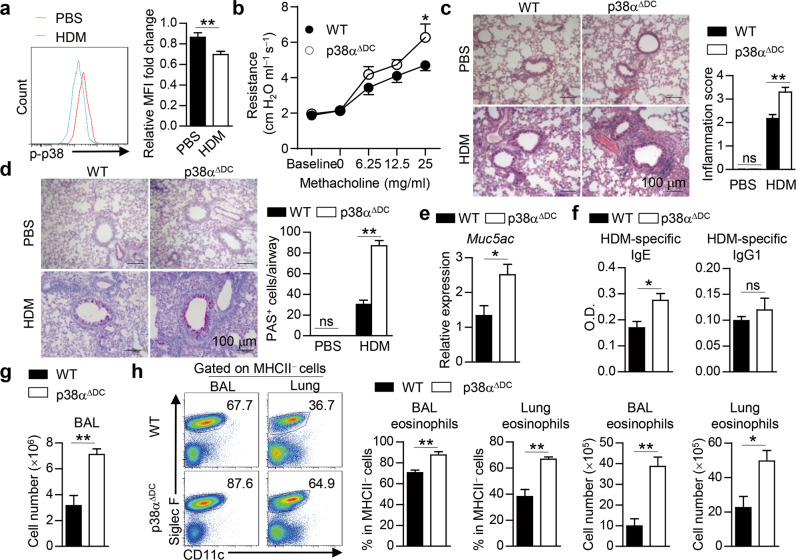
DC-specific p38α deletion renders mice susceptible to HDM-induced allergic asthma. **a** WT mice were *i.n*. sensitized with HDM for 24 h, and the activity of p38 in lung DCs (CD11c^+^MHCII^high^) was examined by flow cytometry and is presented relative to that of lung DCs from PBS-treated mice, which was set as 1 (*n* = 11–14).**b**–**h** WT and p38α^ΔDC^ mice were sensitized with HDM on Days 0–2 and challenged with HDM on Days 14–16. Mice were sacrificed for analysis on Day 17 (**b**, *n* = 7; **c**–**h**, *n* = 4).Airway resistance was measured (**b**). H&E (**c**) and PAS (**d**) staining of lung sections and quantification. Scale bars represent 100 μm. The expression of *Muc5ac* in lung tissues was detected by qPCR (**e**). Serum HDM-specific IgE and IgG1 were detected by ELISA (**f**). Total cell number in the BALF (**g**). The percentage and cell number of eosinophils were detected by flow cytometry (**h**). *P < 0.05; **P < 0.01; ns, not significant. Data are pooled from four experiments (**a**) or representative of two (**b**) or three (**c**–**h**) independent experiments with consistent results. Student’s *t* test (**a**, **e**–**h**) or two*-*way ANOVA (**b**–**d**) was performed, and the data are presented as the mean ± SEM

DCs play an essential role in asthma pathogenesis [91]. We observed a substantial increase in DC numbers both in the lungs and in the bronchial alveolar lavage fluid (BALF) of mice after HDM treatment (Fig. S1a, b). However, p38 activity, as indicated by the level of phosphorylated (p-) p38, in lung DCs from HDM-treated mice was relatively low (Fig. 1a), indicating a potential function for p38α signaling in DCs in the pathogenesis of HDM-induced asthma. To explore the role of DC-intrinsic p38α signaling in asthma pathogenesis, we generated mice with specific p38α deletion in DCs by crossing *Mapk14* (encoding p38α) ^fl/fl^ mice with CD11c-Cre mice, referred to as p38α^ΔDC^ mice [61]. We observed efficient deletion of p38α in the lung and splenic DCs of p38α^ΔDC^ mice (Fig. S1c, d). Importantly, p38α deletion in DCs did not affect the percentage, subsets or activation status of lung DCs (Fig. S1e, f) or homeostasis of other immune cells in the lungs (Fig. S1g, h).

Wild-type (WT) and p38α^ΔDC^ mice were sensitized and challenged by *intranasal* (*i.n*.) administration of HDM to induce asthma (Fig. S2a). We found that p38α^ΔDC^ mice had much higher airway responsiveness to methacholine stimulation (Fig. 1b).Histological analysis showed that the lungs of p38α^ΔDC^ mice had higher inflammatory cell infiltration and mucus secretion, as shown by hematoxylin and eosin (H&E) and periodic acid-Schiff (PAS) staining, respectively (Fig. 1c, d). The expression of *Muc5ac*, which encodes goblet cell mucin, was also increased in the lungs of p38α^ΔDC^ mice (Fig. 1e). In addition, the serum HDM-specific IgE level was increased in p38α^ΔDC^ mice, while the serum HDM-specific IgG1 level was comparable between the two mouse strains (Fig. 1f). To evaluate pulmonary inflammation, we counted the total cell number and examined the cellular composition in the BALF by flow cytometry. The results showed that the total cell number in the BALF of p38α^ΔDC^ mice was much higher than that in the BALF of WT mice (Fig. 1g). Flow cytometry results showed increased eosinophils and comparable neutrophils in the BALF and lungs between p38α^ΔDC^ and WT mice (Fig. 1h and Fig. S2b–d). Moreover, macrophage, DC, CD4^+^ T-cell and CD4^−^ T-cell infiltration was comparable between WT and p38α^ΔDC^ mice (Fig. S2e–g). Thus, mice deficient in p38α in DCs are highly susceptible to HDM-induced experimental allergic asthma, suggesting an important role for p38α in DCs in regulating allergic inflammation.

### p38α deletion in DCs promotes Th2 priming during the sensitization phase but not the effector phase

**Fig. 2:**
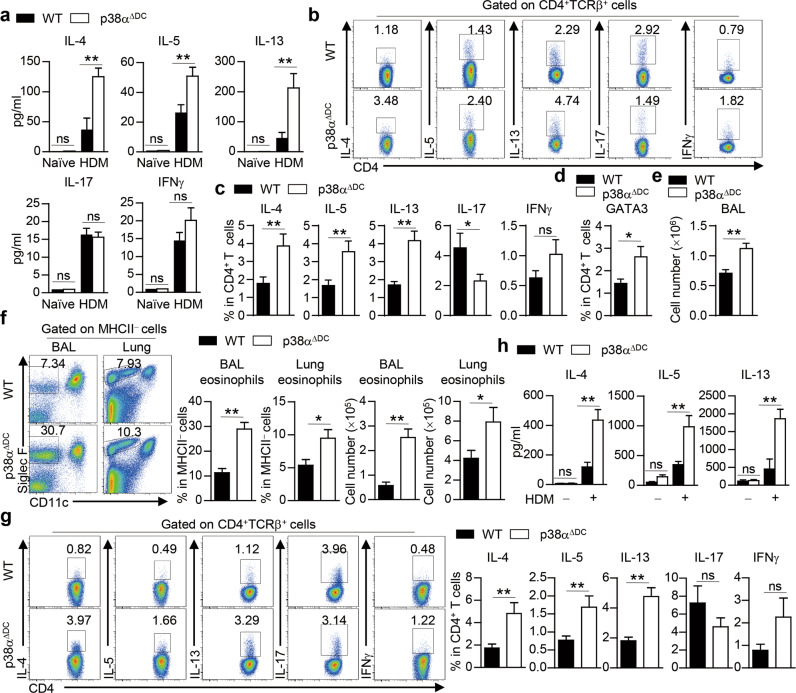
p38α deletion in DCs promotes Th2 priming and allergic inflammation during the sensitization phase. **a**–**c** WT and p38α^ΔDC^ mice were sensitized with HDM on Days 0–2 and challenged with HDM on Days 14–16. Mice were sacrificed for analysis on Day 17. ELISA analysis of IL-4, IL-5, IL-13, IL-17 and IFNγ production in the BALF (**a**) (*n* = 3–4). The percentages of IL-4^+^, IL-5^+^, IL-13^+^, IL-17^+^ and IFNγ^+^ cells in CD4^+^ T cells were determined by intracellular staining (**b**, **c**) (*n* = 13). Percentages of GATA3^+^ cells in CD4^+^ T cells (**d**) (*n* = 5). **e**–**h** WT and p38α^ΔDC^ mice were sensitized with HDM for 3 days and analyzed 7 days later. Total cell number in the BALF (**e**) (*n* ≥ 14). The percentage and cell number of eosinophils were detected by flow cytometry (**f**) (*n* ≥ 14). The percentages of IL-4^+^, IL-5^+^, IL-13^+^, IL-17^+^ and IFNγ^+^ cells in CD4^+^ T cells were determined by intracellular staining (**g**) (*n* ≥ 19). ELISA analysis of ex vivo-isolated mLN cells restimulated with or without HDM for 72 h (**h**) (*n* = 3–4). *P < 0.05; **P < 0.01; ns, not significant. Data are representative of three (**a**, **d**, **h**) independent experiments or pooled from three (**b**–**c**, **e** and **f**) or four (**g**) experiments with consistent results. Student’s *t* test (**c**–**g**) or two-way ANOVA (**a** and **h**) was performed, and the data are presented as the mean ± SEM

In addition to evaluating airway inflammation, we examined the expression of certain proinflammatory cytokines. Upon HDM challenge, the production of certain type 2 cytokines, such as IL-4, IL-5 and IL-13, in the BALF was higher in p38α^ΔDC^ mice than in WT mice, while the IL-17 and IFNγ levels were comparable (Fig. 2a). Flow cytometry assays showed that the increased IL-4, IL-5 and IL-13 production in the lungs of p38α^ΔDC^ mice was mainly from CD4^+^ T cells (Fig. 2b, c), not from CD4^−^ cells (Fig. S3a). The expression of the Th2-specific transcription factor GATA3 was also increased in lung CD4^+^ T cells (Fig. 2d). Thus, p38α signaling in DCs mainly affects Th2-cell generation during asthma pathogenesis.

HDM-induced allergic asthma exhibits sensitization and effector phases [92]. To explore whether p38α signaling in DCs plays a role in the allergic sensitization phase, we sensitized mice with HDM for 3 days and analyzed them 7 days later, which recapitulated the early features of allergic asthma [93]. The total number of cells was increased in the BALF of p38α^ΔDC^ mice (Fig. 2e). Compared to WT mice, p38α^ΔDC^ mice exhibited higher eosinophil infiltration in the BALF and lungs (Fig. 2f). There was higher expression of *Il4*, *Il5*, *Il13* and *Gata3* but lower expression of *Il17* in lung tissues from p38α^ΔDC^ mice, while the expression of *Ifng* and *Il9* was similar between the mouse strains (Fig. S3b). Intracellular staining showed that IL-4, IL-5 and IL-13 production by lung CD4^+^ T cells was higher in p38α^ΔDC^ mice, while IL-17 and IFNγ production was unaffected (Fig. 2g). The increased levels of Th2 cytokines in p38α^ΔDC^ mice could be due to enhanced T-cell proliferation or survival. To explore whether p38α deficiency in DCs affects T-cell proliferation and survival, we analyzed Ki67 and active Caspase-3 expression. We found that WT and p38α^ΔDC^ mice had comparable Ki67 and active Caspase-3 staining in CD4^+^ T cells isolated from the lungs following HDM treatment (Fig. S3c, d). We also analyzed T-cell responses in the mLNs by ex vivo restimulation of mLN cells with HDM. mLN cells from p38α^ΔDC^ mice had higher IL-4, IL-5 and IL-13 production (Fig. 2h), indicating that p38α deletion in DCs promotes Th2 priming. Group 2 innate lymphoid cells (ILC2s) have been shown to be essential in Th2 cell-mediated allergic lung inflammation during the early stage of sensitization [94, 95,]. Our results showed that p38α activity in DCs did not affect type-2 cytokine secretion by ILCs in HDM-sensitized mice (Fig. S3e). Papain has been reported to rapidly induce ILC2s [96]. WT and p38α^ΔDC^ mice had comparable IL-4 and IL-13 production by ILC2s in a papain-induced acute lung inflammation model (Fig. S3f). These results suggest that p38α in DCs plays an important role in the regulation of HDM-induced Th2 priming and Th2-dependent immune responses during the sensitization phase.

To explore whether p38α signaling in DCs also plays a role in the effector allergic phase, we transferred HDM-pulsed WT or p38α^ΔDC^ bone morrow-derived DCs (BMDCs) into HDM-sensitized WT mice (Fig. S4a). The recipients transferred with WT or p38α-deficient BMDCs had comparable eosinophil infiltration in both the lungs and the BALF (Fig. S4b), as well as comparable IL-4, IL-5, IL-13 and IFNγ production in lung CD4^+^ T cells (Fig. S4c, d). To further examine whether p38α signaling in DCs affects effector Th2-cell generation, we transferred mLN cells isolated from HDM-sensitized CD45.1 mice into WT and p38α^ΔDC^ CD45.2 mice and then challenged the recipients with HDM, followed by analysis of donor effector CD4^+^ T-cell development (Fig. S4e). Donor CD4^+^ T cells isolated from WT or p38α^ΔDC^ recipients produced comparable levels of IL-4, IL-5 and IL-13 in the lungs (Fig. S4f). Thus, p38α signaling programs DCs to regulate Th2-mediated allergic inflammation during the sensitization phase, not the effector phase.

### p38α signaling in cDC1s regulates Th2-cell differentiation in vitro and in vivo

**Fig. 3:**
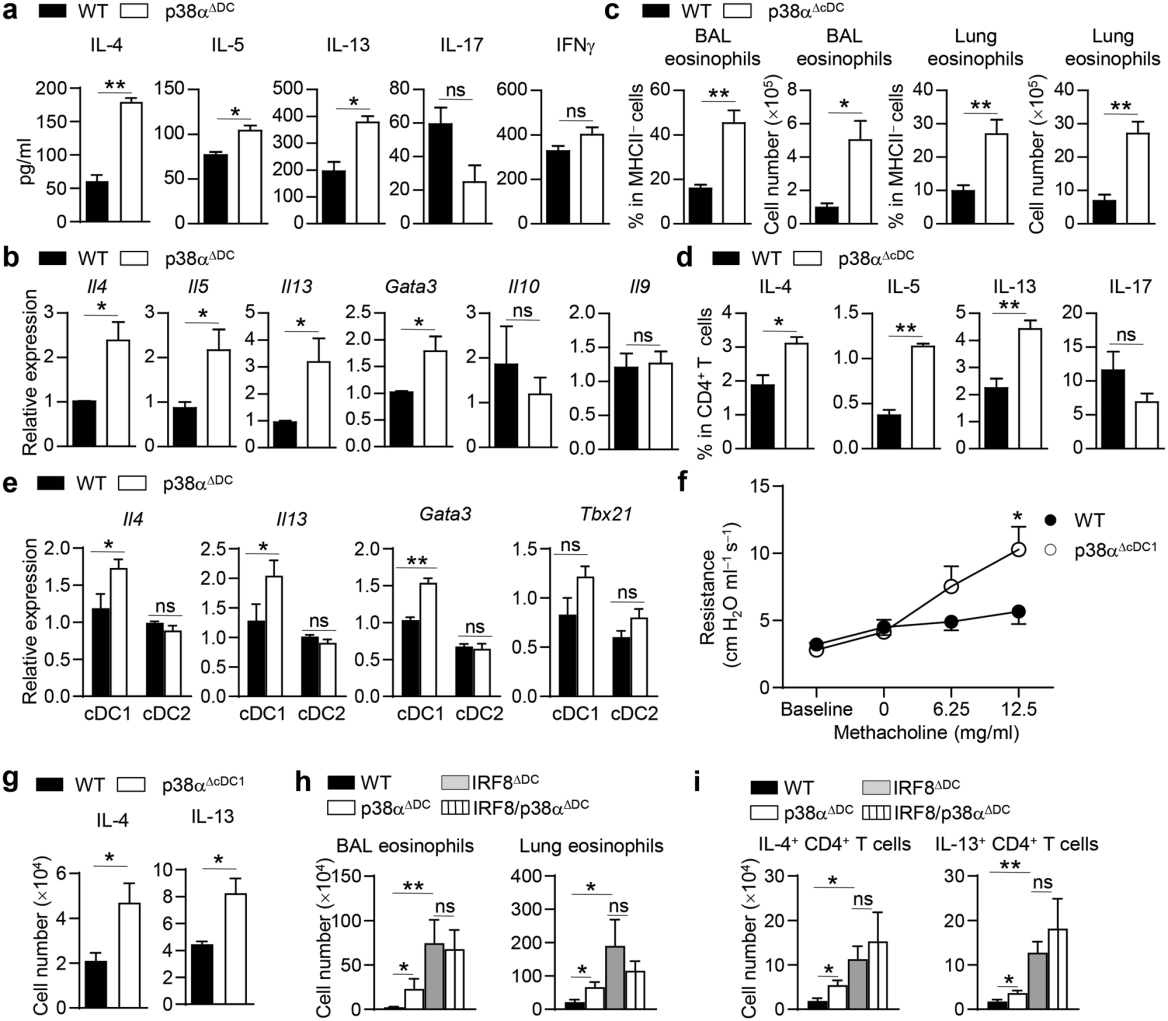
p38α signaling in cDC1s regulates Th2 responses upon HDM treatment. **a** Flow cytometry-sorted lung DCs from HDM-treated WT and p38α^ΔDC^ mice were cocultured with naïve OT-II CD4^+^ T cells in the presence of OVA_323-339_ for 72 h. ELISA analysis of IL-4, IL-5, IL-13, IL-17 and IFNγ production (*n* = 3). **b** Analysis of *Il4*, *Il5*, *Il13*, *Gata3*, *Il10 and Il9* mRNA expression in OT-II CD4^+^ T cells (*n* = 4). **c**, **d** WT and p38α^ΔcDC^ mice were sensitized and challenged with HDM to induce asthma (*n* = 3). Percentages and cell numbers of eosinophils in the BALF and lung tissues (**c**). Percentages of IL-4, IL-5, IL-13 and IL-17 in lung CD4^+^ T cells (**d**). **e** mRNA expression of *Il4*, *Il13*, *Gata3* and *Tbx21* in OT-II CD4^+^ T cells activated by WT or p38α-deficient lung cDC1 or cDC2 subsets from HDM-treated mice in the presence of OVA_323-339_ (*n* = 2–3, 1 DC subset sample pooled from at least 3 mice). **f** and **g** WT and p38α^ΔcDC1^ mice were sensitized and challenged with HDM to induce asthma (*n* = 3–7). Airway resistance was measured (**f**). The cell numbers of IL-4^+^CD4^+^ T cells and IL-13^+^CD4^+^ T cells in lung tissues were measured (**g**). **h** and **i** WT, p38α^ΔDC^, IRF8^ΔDC^ and IRF8/p38α^ΔDC^ mice were sensitized and challenged with HDM to induce asthma (*n* = 3–6). The numbers of eosinophils in the BALF and lung tissues (**h**) and the numbers of IL-4^+^CD4^+^ T cells and IL-13^+^CD4^+^ T cells in lung tissues were measured (**i**). *P < 0.05; **P < 0.01; ns, not significant. Data are pooled from three (**a** and **b**) or two (**h** and **i**) experiments with consistent results or representative of three (**c**–**e**) or two (**f** and **g**) experiments with consistent results. Student’s *t* test (**a**–**d**, **g**–**i**) or two-way ANOVA (**e** and **f**) was performed, and the data are presented as the mean ± SEM

To determine whether p38α mediates the crosstalk between lung DCs and CD4^+^ T cells by driving the lineage differentiation of antigen-specific naïve precursors, we cocultured naïve OT-II CD4^+^ T cells with lung DCs isolated from HDM-sensitized WT or p38α^ΔDC^ mice in the presence of OVA_323-339_ in vitro. OT-II CD4^+^ T cells activated with p38α-deficient lung DCs exhibited higher *Il4*, *Il5*, *Il13* and *Gata3* expression at the mRNA and/or protein levels, while the expression of *Il17*, *Ifng*, *Il10* and *Il9* was comparable (Fig. 3a, b). The proliferation and survival of CD4^+^ T cells were not affected (Fig. S5a, b). These results show that p38α signaling in DCs is important for the regulation of Th2-cell differentiation in vitro.

To further explore the role of DC subset-intrinsic p38α signaling in asthma pathogenesis, we generated mice with specific p38α deletion in cDCs by crossing *Mapk14*^fl/fl^ mice with *zbtb46*-Cre mice [97], referred to as p38α^ΔcDC^ mice. We observed efficient deletion of p38α in spleen cDCs but not in the pDCs of p38α^ΔcDC^ mice (Fig. S6a). WT and p38α^ΔcDC^ mice were sensitized and challenged by *i.n*. administration of HDM to induce asthma. While p38α deletion in cDCs did not affect the percentage, subsets or activation status of lung cDCs (Fig. S6b, c), the infiltration of eosinophils into the BALF and lungs was increased in p38α^ΔcDC^ mice (Fig. 3c). Th2 cytokine expression was also increased in lung tissues of p38α^ΔcDC^ mice (Fig. 3d). These results demonstrate that p38α signaling in cDCs regulates HDM-induced allergic inflammation.

To examine whether p38α signaling in different lung cDC subsets has different abilities to regulate Th2-cell differentiation, we first isolated lung cDC1s (CD19^−^Siglec F^−^CD11c^+^MHCII^+^CD103^+^CD11b^−^) and cDC2s (CD19^−^Siglec F^−^CD11c^+^MHCII^+^ CD103^−^CD11b^+^) from HDM-sensitized WT and p38α^ΔDC^ mice for RNA-seq analysis. Pearson correlation and clustering of the sample groups confirmed that the transcriptomes of cDC1s and cDC2s were distinct and suggested that the effects of p38α deficiency under HDM stress were more pronounced in the cDC1 population (Fig. S7a). A principal component analysis plot of the RNA-seq results revealed that p38α-deficient cDC1s developed a highly distinct transcriptome following HDM sensitization. The variation in PC1 was predominantly explained by the cells being either cDC1s or cDC2s, while that in PC2 was mostly explained by p38α deficiency (Fig. S7b). This was also confirmed by the differentially expressed genes determined from the RNA-seq results (Dataset S1). To further confirm the RNA-seq results, we cocultured WT and p38α-deficient lung cDC1s or cDC2s with naïve OT-II CD4^+^ T cells for cytokine analysis. Compared to WT lung cDC1s, p38α-deficient lung cDC1s induced higher *Il4*, *Il13* and *Gata3* expression and comparable *Tbx21* expression, while p38α was not required for lung cDC2-driven Th2-cell differentiation (Fig. 3e). To evaluate the importance of p38α in lung cDC1s in regulating HDM-induced Th2 immunity in vivo, we generated cDC1-specific p38α knockout mice by crossing *Xcr1*-Cre mice [71] with *Mapk14*^fl/fl^ mice, referred to as p38α^ΔcDC1^ mice. We observed efficient deletion of p38α in the splenic cDC1s of p38α^ΔcDC1^ mice (Fig. S8a). p38α deletion in cDC1s did not affect the percentage of lung DC subsets (Fig. S8b). After HDM treatment, we found that p38α^ΔcDC1^ mice had much higher airway responsiveness to methacholine stimulation than WT mice and increased IL-4 and IL-13 production in lung CD4^+^ T cells (Fig. 3f, g). We also used an *Irf8*-deficient mouse line (*Irf8*^fl/fl^CD11c-cre mice) and an *Irf4*-deficient mouse line (*Irf4*^fl/fl^CD11c-cre mice) to specifically target CD11c-expressing cDC1s and cDC2s in vivo, respectively [70]. By crossing *Mapk14*^fl/fl^ mice with *Irf8*^fl/fl^CD11c-cre mice or *Irf4*^fl/fl^CD11c-cre mice, we explored the roles of p38α in cDC1s and cDC2s in vivo. We analyzed the DC population in lung tissues of WT, p38α^ΔDC^, IRF8^ΔDC^ and IRF8/p38α^ΔDC^ mice and found that the percentage of total DCs was comparable among these four strains of mice, but the percentage of cDC1s was decreased in the lungs in IRF8^ΔDC^ and IRF8/p38α^ΔDC^ mice (Fig. S8c). After the establishment of HDM-induced asthma, we found that although the infiltration of eosinophils into the BALF and lungs and that of IL-4^+^CD4^+^ T cells and IL-13^+^CD4^+^ T cells into the lungs were higher in IRF8^ΔDC^ mice than in WT mice, infiltration was comparable between IRF8^ΔDC^ mice and IRF8/p38α^ΔDC^ mice (Fig. 3h, i). We also established an HDM-induced asthma model with WT, p38α^ΔDC^, IRF4^ΔDC^ and IRF4/p38α^ΔDC^ mice. The infiltration of eosinophils and IL-4^+^CD4^+^ T cells into lung tissues of IRF4^ΔDC^ mice was lower than that into lung tissues of WT mice. Deletion of cDC2s in p38α^ΔDC^ mice (IRF4/p38α^ΔDC^) increased the infiltration of eosinophils, IL-4^+^CD4^+^ T cells and IL-13^+^CD4^+^ T cells into lung tissues compared with the deletion in IRF4^ΔDC^ mice (Fig. S9a, b). Collectively, these data indicate that p38α signaling in cDC1s but not in cDC2s impacts Th2-cell differentiation upon HDM stimulation.

### p38α signaling in cDC1s regulates Th2-cell differentiation by modulating IL-12 expression

**Fig. 4:**
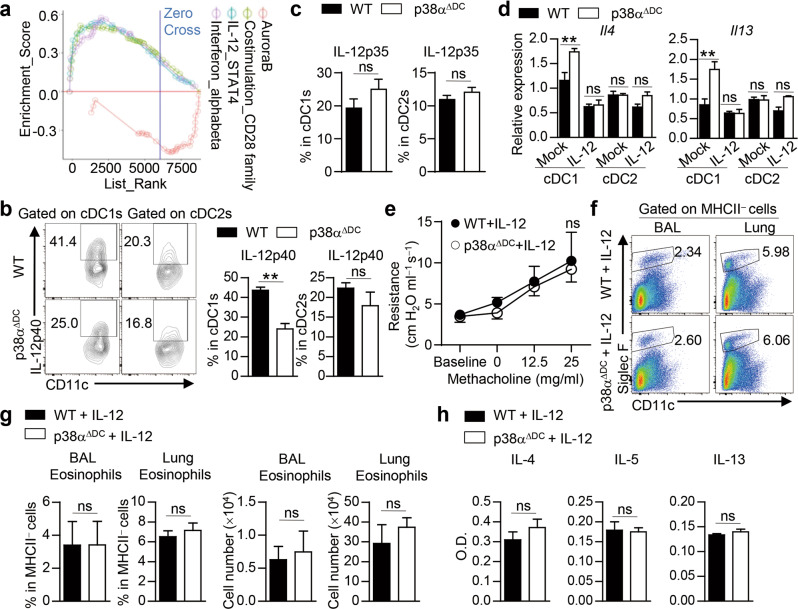
p38α signaling in lung cDC1s regulates Th2-cell differentiation by modulating IL-12 expression. **a** GSEA of cDC1s. **b** IL-12p40 and (**c**) IL-12p35 expression in HDM-treated WT and p38α-deficient lung DC subsets analyzed by flow cytometry (*n* = 4). **d** mRNA expression of *Il4* and *Il13* in CD4^+^ T cells activated with WT or p38α-deficient lung cDC1 or cDC2 subsets from HDM-treated mice with or without IL-12 for 3 days (*n* = 2–3, 1 DC subset sample pooled from at least three mice). **e**–**h** WT and p38α^ΔDC^ mice were sensitized with HDM on Days 0–2 and challenged with HDM on Days 14–16, and then IL-12p70 was *i.n*. administered during HDM treatment. Mice were sacrificed for analysis on Day 17 (*n* = 3–4). Airway resistance was measured (**e**). Eosinophil infiltration in the BALF and lung tissues was detected by flow cytometry and quantified (**f** and **g**). The concentrations of IL-4, IL-5 and IL-13 in the BALF were detected by ELISA (**h**). **P < 0.01; ns, not significant. Data are representative of three (**b**–**d** and **f**–**h**) or two (**e**) independent experiments. Student’s *t* test (**b**, **c**, **g** and **h**) or two-way ANOVA (**d** and **e**) was performed, and the data are presented as the mean ± SEM

Next, we explored the underlying molecular mechanisms by which p38α regulates lung DC function to shape Th2-cell differentiation. Gene set enrichment analysis (GSEA) of lung cDC1s demonstrated that HDM challenge led to enrichment of costimulatory factors and cytokine signaling molecules (Fig. 4a). IL-12 has been reported to suppress Th2-cell differentiation [98]. We found decreased IL-12p40 expression in p38α-deficient lung cDC1s from HDM-sensitized mice (Fig. 4b), and the expression of IL-12p35 was comparable between WT and p38α^ΔDC^ mice (Fig. 4c). To explore whether the decreased IL-12p40 expression in p38α-deficient lung cDC1s could contribute to enhanced Th2-cell differentiation, we added IL-12 to DC−T-cell coculture systems. The addition of IL-12 reduced the IL-4 and IL-13 levels in T cells activated by p38α-deficient lung cDC1s to the levels induced by WT lung cDC1s, but it did not affect p38α-deficient lung cDC2-activated T cells (Fig. 4d). To further evaluate the role of IL-12 in p38α-dependent Th2 responses in vivo, we injected IL-12 *i.n*. into WT and p38α^ΔDC^ mice during HDM treatment and found that the airway responsiveness to methacholine, infiltration of eosinophils in the BALF and lungs and concentrations of IL-4, IL-5 and IL-13 in the BALF were comparable between WT and p38α^ΔDC^ mice upon IL-12 injection (Fig. 4e–h). Notably, IL-12 treatment did not change eosinophil infiltration or Th2 cytokine production in non-HDM-treated WT mice but largely decreased eosinophil infiltration and Th2 cytokine production upon HDM treatment. However, IL-12 treatment abrogated the enhancing effects of HDM on the Th2 cytokine production and eosinophil infiltration in WT mice (Fig. S10a, b). Collectively, these data indicate that p38α signaling regulates lung cDC1-dependent Th2-cell differentiation by modulating IL-12 expression.

### p38α regulates IL-12 expression by modulating the MK2−c-FOS axis in DCs

**Fig. 5:**
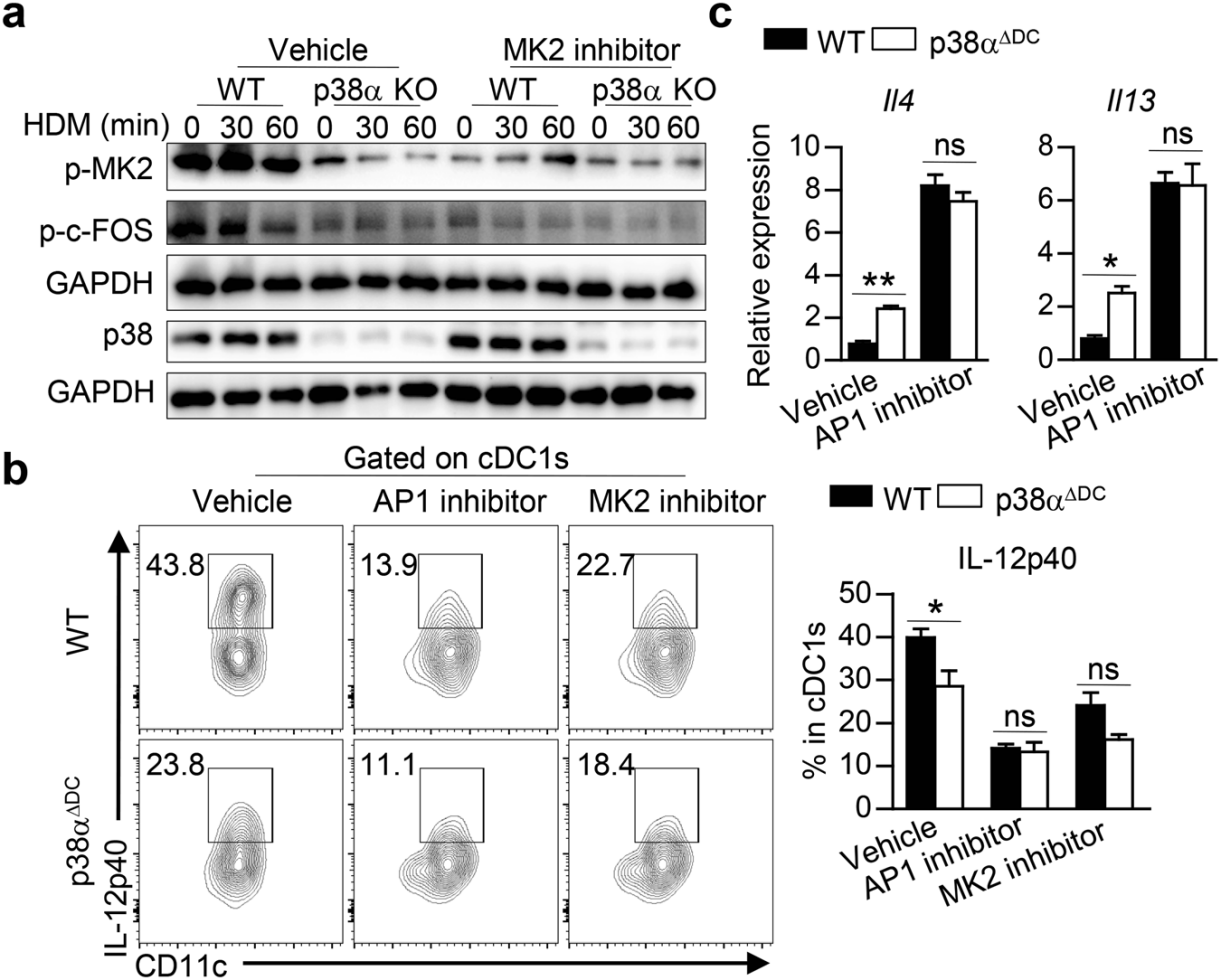
p38α regulates IL-12 expression by modulating the MK2−c-FOS signaling pathway axis in DCs. **a** The abundances of p-MK2, p-c-FOS and p38 in Flt3L-DCs stimulated with HDM in the presence of vehicle or an MK2 inhibitor were analyzed by Western blot analysis. **b** Percentages of IL-12p40^+^ cells in HDM-stimulated WT and p38α-deficient lung cDC1s pretreated with vehicle, an AP1 inhibitor or an MK2 inhibitor (*n* = 3). **c** Expression of *Il4* and *Il13* in OT-II CD4^+^ T cells activated with HDM-pretreated Flt3L-cDC1s stimulated with vehicle or an AP1 inhibitor and washed (*n* ≥ 3). *P < 0.05; **P < 0.01; ns, not significant. Data are representative of three (**a**, **b**) independent experiments with consistent results or pooled from two (**c**) independent experiments with consistent results. Two-way ANOVA (**b**, **c**) was performed, and the data are presented as the mean ± SEM

Having shown that p38α signaling in lung cDC1s regulates Th2 differentiation by modulating IL-12 expression, we next examined the signaling and transcriptional mechanisms involved in the regulation of IL-12 expression. Due to the limited number of lung DCs available for Western blot analysis and decreased *Il12p40* expression in HDM-stimulated p38-deficient Flt3L-derived BMDCs (Flt3L-DCs) (Fig. S10c), we used Flt3L-DCs for further Western blot analysis. MAPK-activated protein kinase 2 (MK2) is the essential downstream component of p38, and p38-MK2 signaling has been reported to play different roles in regulating IL-12 secretion in different human DC subsets [99]. In our study, the phosphorylation level of MK2 was decreased in HDM-treated p38α-deficient Flt3L-DCs, and inhibition of MK2 activity reduced IL-12p40 expression in p38α-deficient lung cDC1s to the level in WT cDC1s (Fig. 5a, b). Next, we explored the mechanism underlying the regulation of IL-12p40 transcription by p38α in DCs. It has been reported that the production of IL-12 is increased in LPS-stimulated c-FOS-deficient macrophages [100]. In our study, we found that p-c-FOS was decreased in p38α-deficient Flt3L-DCs upon HDM stimulation and that inhibition of c-FOS activity with an AP1 inhibitor further reduced IL-12p40 expression to the same level as that in WT DCs, while activation of AP-1 by EGF stimulation led to increased IL-12p40 expression in p38α-deficient DCs comparable to the level in WT DCs (Fig. 5a, b, and Fig. S10d). The expression of IL-4 and IL-13 in OT-II CD4^+^ T cells activated with WT or p38α-deficient Flt3L-DCs was also increased to a comparable level upon AP1 inhibition (Fig. 5c). Moreover, MK2 inhibition decreased c-FOS activity in both WT and p38α-deficient Flt3L-DCs to a similar level (Fig. 5a). Collectively, these results demonstrate that p38α regulates IL-12 expression by modulating the MK2−c-FOS axis in DCs.

### p38α activity in lung alveolar macrophages is not required for Th2-mediated allergic lung inflammation

AMs are one of the most prominent immune cells in the airways. They are known to regulate pro- and anti-inflammatory responses in the airways and play a critical role in asthma pathogenesis [101]. AMs share the common surface marker CD11c with DCs. To determine the role of p38α signaling in AMs in mediating Th2-dependent allergic inflammation, we generated *Mapk14*^fl/fl^ LysM-Cre mice, referred to as p38α^ΔLysM^ mice, in which p38α is deleted exclusively in mature macrophages and monocyte-derived cells [102]. We observed efficient deletion of p38α in AMs (Fig. S11a). p38α deletion in macrophages did not affect the percentages of lung macrophages or DCs (Fig. S11b). Upon HDM treatment, WT and p38α^ΔLysM^ mice had comparable neutrophil and eosinophil infiltration in the BALF and lungs (Fig. S11c, d), as well as Th2 cytokine expression in lung tissues (Fig. S11e). These results demonstrate that p38α signaling in AMs is not required for allergic inflammation.

### DCs integrate allergic instructive signals and Th2-polarized cytokine signals via p38α to regulate Th2-cell differentiation

**Fig. 6:**
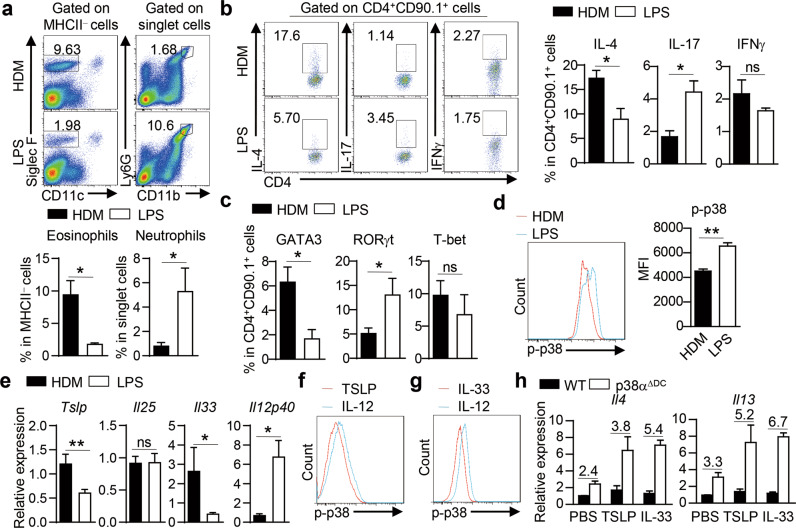
DCs integrate allergic instructive signals and Th2-polarized cytokine signals via p38α to regulate Th2-cell differentiation. **a**, **b** A total of 2 × 10^6^ naïve OT-II CD4^+^ T cells were *i.v*. transferred into C57BL/6 mice on day −1, and then the mice were *i.n*. treated with OVA plus HDM or LPS on Day 0. Mice were analyzed on Day 7 (*n* = 4–6). Flow cytometric analysis of eosinophils and neutrophils in the BALF (**a**). IL-4, IL-17, and IFNγ (**b**) or GATA3, RORγt and T-bet expression (**c**) in donor OT-II CD4^+^ T cells in lung tissues. **d** Flow cytometric analysis of p38 activity in HDM- or LPS-stimulated lung DCs (*n* = 6–7). **e** WT mice were *i.n*. immunized with 10 ng LPS or 50 μg HDM for 3 days, and the mRNA expression of *Tslp*, *Il25*, *Il33* and *Il12p40* was measured by qPCR (*n* ≥ 3). **f**, **g** Flow cytometric analysis of p38 activity in TSLP-, IL-33- or IL-12-stimulated DCs. **h** RNA analysis of *Il4* and *Il13* in OT-II CD4^+^ T cells activated with WT or p38α-deficient lung DCs in the presence of 10 ng/ml IL-33 or 10 ng/ml TSLP. The numbers above the bars indicate the ratio of *Il4* or *Il13* mRNA in T cells stimulated with p38α-deficient DCs to that in T cells stimulated with WT DCs (*n* ≥ 3). *P < 0.05; **P < 0.01; ns, not significant. Data are pooled from two experiments with consistent results (**a**, **e** and **h**) or representative of two (**b** and **c**) or three (**d**, **f** and **g**) independent experiments. Student’s *t* test (**a**–**e**) was performed, and the data are presented as the mean ± SEM

We next investigated the upstream signals that regulated p38α activation in DCs to ‘instruct’ Th2-cell differentiation. In an in vivo antigen-specific OVA challenge model, we showed that HDM induced a higher Th2 response and more pronounced eosinophil-dominant allergic lung inflammation, while LPS induced a strong Th17 response and high neutrophil infiltration in the lungs (Fig. 6a–c). These changes were associated with lower p38 activity induction in DCs by HDM than by LPS (Fig. 6d). Considering that LPS is the most commonly used adjuvant to induce inflammatory signals and HDM is an allergic signal inducer, our results indicate that allergic instructive signals induce lower p38 activity in DCs, which facilitates a strong Th2 response.

In addition to responding to environmental stimuli directly, DCs can be indirectly activated by other cells or cytokines in the local environment [33]. Barrier ECs are the first line of defense and can mount a prototypical response by releasing Th2-instructive cytokines, such as IL-25, IL-33 and TSLP. These signals are thought to play a pivotal role in conditioning DC activation and function [33, 103,]. The expression of IL-33 and TSLP was higher in HDM-treated lung tissues than in LPS-treated lung tissues, while IL-25 was comparable, but LPS induced higher IL-12p40 expression than did HDM (Fig. 6e). Next, we measured the activity of p38 in DCs stimulated with TSLP, IL-33 or IL-12 and found that TSLP and IL-33 induced lower p-p38 levels than IL-12 (Fig. 6f, g), indicating that Th2-instructive signals bias DCs toward lower p38 activity. While exogenous TSLP or IL-33 had a modest effect on IL-4 and IL-13 expression in T cells activated by WT DCs, they dramatically increased the expression of IL-4 and IL-13 in T cells activated by p38α-deficient DCs (Fig. 6h). Collectively, these results indicate that DCs integrate both allergic and Th2-polarized cytokine signals *via* p38α to regulate Th2-cell differentiation.

### Deletion of p38α in DCs promotes FITC-induced contact hypersensitivity (CHS) responses and antiparasite immune responses

**Fig. 7:**
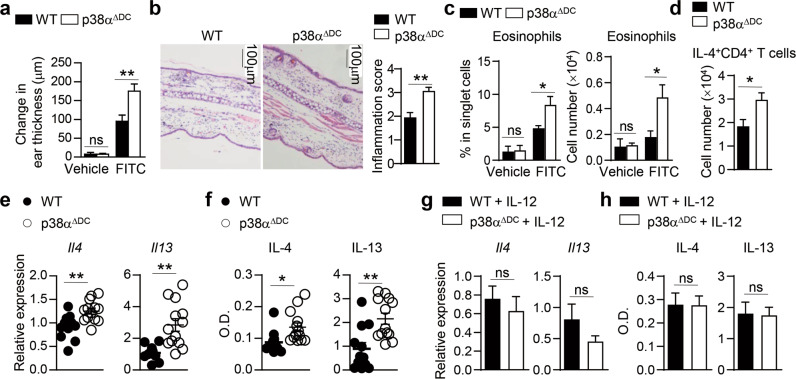
Deletion of p38α in DCs promotes FITC-induced skin contact hypersensitivity and antiparasite immunity. **a**–**d** CHS was induced in WT and p38α^ΔDC^ mice with FITC. Change in ear thickness (**a**) (*n* = 4). Histological analysis of ear sections and quantification. Scale bars represent 100 μm (**b**) (*n* = 4). Percentage and cell number of eosinophils in the ear (**c**) (Vehicle, *n* = 2, 1 sample pooled from 2 mice; FITC, *n* = 4). IL-4^+^CD4^+^ T cells in the skin-draining lymph nodes analyzed by flow cytometry (**d**) (*n* = 5). **e**, **f** WT and p38α^ΔDC^ mice were immunized with *Schistosoma japonicum* eggs (*n* ≥ 11). dLN cells were stimulated with SEA for 48 or 72 h. The cells were harvested for mRNA analysis (**e**), and the supernatant was harvested for ELISA (**f**). **g**, **h** WT and p38α^ΔDC^ mice were immunized with *Schistosoma japonicum* eggs, and IL-12 was injected into the footpad at the time of *S. japonicum* egg injection (*n* = 5). dLN cells were stimulated with SEA for 48 or 72 h. The cells were harvested (48 h) for mRNA analysis (**g**), and the supernatant was harvested (72 h) for ELISA (**h**). *P < 0.05; **P < 0.01; ns, not significant. Data are representative of two independent experiments (**a**–**d**, **g**, **h**) or pooled from three independent experiments with consistent results (**e**, f). Student’s *t* test (**b**, **d**–**h**) or two-way ANOVA (**a**, **c**) was performed, and the data are presented as the mean ± SEM

The combination of the hapten FITC with the phthalate ester dibutyl phthalate (DBP) can induce a robust Th2-dependent CHS response in mice [104, 105,]. To examine whether p38α activity in DCs is generally required for the Th2 response induced by allergic stimulation, we sensitized and challenged WT and p38α^ΔDC^ mice with FITC-DBP. Ear swelling was much more severe in p38α^ΔDC^ mice than in WT mice 24 h after challenge (Fig. 7a). Histological analysis showed that the challenged p38α^ΔDC^ mice had substantial cellular infiltration, epidermal thickening and lesions (Fig. 7b). Flow cytometric analysis showed that the infiltration of eosinophils in the skin of p38α^ΔDC^ mice was much higher than that in the skin of WT mice (Fig. 7c). There were more IL-4-producing CD4^+^ T cells in p38α^ΔDC^ mice than in WT mice (Fig. 7d). These results indicate that allergic Th2 responses in the skin are also regulated by p38α activity in DCs.

Th2 cells are essential for antiparasite immunity [10]. To determine whether p38α signaling is also critical for DC-mediated Th2 responses during antiparasite immunity, we subcutaneously (*s.c*.) injected eggs from *Schistosoma japonicum* into the footpad of WT and p38α^ΔDC^ mice. One week later, we analyzed the immune responses via ex vivo restimulation of draining popliteal LN (dLN) cells with soluble egg antigen (SEA) from *Schistosoma japonicum*. We found that the expression of IL-4 and IL-13 was increased in the cells from p38α^ΔDC^ mice at both the mRNA and protein levels (Fig. 7e, f). We injected IL-12 into the footpad of WT and p38α^ΔDC^ mice at the time of *S. japonicum* egg injection. The results showed that IL-12 treatment was sufficient to limit the exacerbated Th2 response induced in p38α^ΔDC^ mice (Fig. 7g, h). These results demonstrate that Th2-dependent CHS and antiparasite immunity are generally regulated by p38α signaling in DCs.

### Inhibition of p38α in human DCs enhances Th2-cell differentiation

**Fig. 8:**
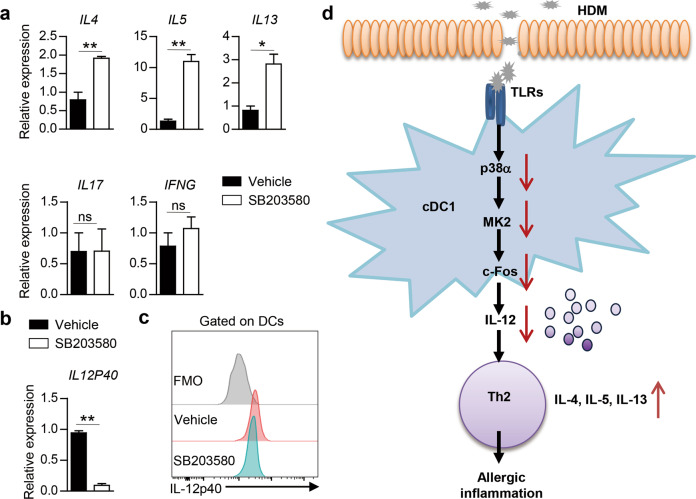
Inhibition of p38 activity in human DCs promotes Th2-cell differentiation. **a** RNA analysis of *IL4*, *IL5*, *IL13*, *IL17* and *IFNG* in human blood CD4^+^ T cells activated with human mo-DCs pretreated with HDM in the presence of vehicle or SB203580 (*n* = 3). **b** Expression of *IL12P40* in human mo-DCs stimulated with HDM in the presence of vehicle or SB203580 for 5 h (*n* = 3). **c** Intracellular staining analysis of IL-12p40 expression in AR patient-derived DCs treated with vehicle or SB203580. **d** Proposed model of the involvement of DC p38α signaling in the regulation of Th2-mediated allergic inflammation. *P < 0.05; **P < 0.01; ns, not significant. Data are pooled from three independent experiments (**a**) or representative of three independent experiments (**b**). Student’s *t* test (**a** and **b**) was performed, and the data are presented as the mean  ± SEM

To explore whether p38 signaling in human DCs affects human CD4^+^ T-cell differentiation under allergic instructive conditions, we pretreated DCs derived from human peripheral blood monocytes with the p38 inhibitor SB203580 or vehicle prior to HDM stimulation and cocultured them with naïve human CD4^+^ T cells. The inhibition of p38 in human DCs increased the expression of *IL4*, *IL5* and *IL13* in DC-activated human CD4^+^ T cells without affecting the expression of *IL17* or *IFNG* (Fig. 8a). The inhibition of p38 also decreased IL-12p40 expression in human DCs (Fig. 8b). To further illustrate the links among p38 activity, IL-12 expression and allergic inflammation, PBMCs were collected from AR patients with disease considered to be driven by Th2 responses, similar to allergic asthma. We treated the isolated PBMCs with SB203580 or vehicle and found that IL-12p40 expression in DCs was decreased upon SB203580 treatment (Fig. 8c). Thus, p38 activity represents an evolutionarily conserved pathway that shapes DC-dependent Th2-cell differentiation under allergic conditions (Fig. 8d).

## Discussion

Growing evidence suggests that DCs are necessary and sufficient to initiate and maintain adaptive Th2 responses to allergens [11, 12,], but the mechanism by which DCs respond to allergic signals to further shape  Th2-cell differentiation and inflammation is not fully understood, which puts us at a disadvantage in designing therapeutics for allergic diseases. Here, we report that p38α integrates diverse allergic, parasitic and Th2-instructive cytokine signals in DCs to direct Th2 responses, thereby establishing a new pathway for DC-dependent programming of Th2-cell differentiation. More importantly, p38α deletion specifically in cDC1s but not in cDC2s or macrophages promoted Th2 responses in the context of HDM stimulation. The activity of p38α was conserved between mouse and human DCs. Our study identified crucial roles for the DC p38α−IL-12−Th2 axis in allergic diseases and antiparasite immunity and might provide an attractive treatment for these diseases.

Chronic allergic diseases such as asthma and atopic dermatitis are mainly mediated by ILC2s and Th2 cells [106], although Th9 cells have also been shown to be involved [107, 108,]. Both ILC2s and Th2 cells share core programming and have similar effector cytokines to mediate the features of allergic asthma [106]. In this study, we found that DC p38α signaling had a unique function in directing Th2-cell differentiation by regulating IL-12 expression, while leaving ILC2 differentiation unaffected during the early stage. Moreover, our study indicated that p38α signaling in DCs was not required for IL-9 production during asthma development. Th2 cells are also important in antiparasite immunity [10]. A recent study showed that the Dectin-1/2-Erk-PGE2-OX40L pathway in DCs played an important role in Th2 polarization during helminth infection [109]. In the current study, we identified p38α signaling as a novel signaling pathway in DCs involved in driving Th2 responses to SEA stimulation, although the potential mechanisms need to be further explored in the future. Taken together, our results implicate p38α acting at the DC−T-cell interface in the control of Th2-cell differentiation during allergic disease development and in antiparasite immunity.

Mouse lung cDCs are a heterogeneous population that consists of cDC1 and cDC2 subsets. Due to differences in mouse models and HDM batches and doses, the roles of these two DC subsets in driving Th2 development remain somewhat controversial [13]. While most studies support crucial roles for cDC2s and mo-DCs in asthma pathogenesis, a role for cDC1s in asthma still cannot be ruled out [38, 44,]. cDC1s frequently project their dendritic extensions between ECs, allowing them to directly sample luminal airway antigens [110]. Once epithelial tight junctions are cleaved by the enzymatic activity of HDM, cDC1s can easily migrate to the lymph nodes and drive T-cell priming, which endows this DC subset with an essential role in initiating the immune response to allergens [110]. cDC2s reside beneath the basement membrane in conducting airways and the lung parenchyma, which endows this subset with an efficient capacity for priming and restimulating effector CD4^+^ T cells in the lungs, thus playing a major role in enhancing the allergic effector phase [110]. Following comprehensive in vitro study combined with in vivo analysis of zDC-Cre, Xcr1-cre, IRF8^fl/fl^, and IRF4^fl/fl^ mice, our results demonstrate that p38α signaling specifically in cDC1s initiates and regulates Th2-cell differentiation and allergic inflammation. Notably, most current functional studies on DC subsets are based on known surface markers and may be insufficient for developing detailed descriptions of their functions, especially under variable pathological conditions. Future unsupervised analysis based on multiomic single-cell analysis will be very helpful for exploring the heterogeneity and function of DCs.

Moreover, it is quite interesting that cDC1s appear to be much more functionally dependent on p38α than do cDC2s based on our current study. A detailed study of the expression patterns of genes downstream of p38/MAPK signaling performed with curated gene lists from more datasets might help to determine the underlying mechanism of the Th2 bias observed under HDM stimulation. We believe future work is necessary to understand the true functional significance of the expression of MAPK downstream genes in cDC1s, and we hope to address this question in greater detail in our future investigations. Taken together, these results demonstrate that the division of labor between these two lung DC subsets in the HDM-induced mouse asthma model is mostly regulated by p38α signaling. p38 MAPK plays a central role in the regulation of numerous proinflammatory responses and disease models [111–113]. Our previous studies also demonstrate a crucial role for p38α signaling in DCs in driving diverse T-cell fates and functions [61–64]. Not surprisingly, p38 has been one of the most extensively studied drug targets in the treatment of inflammatory diseases, but clinical trials on several p38 inhibitors have been halted due to severe side effects. Thus [113], detailed mechanistic studies of the function of p38 remain an active and important area of investigation, which would facilitate the development of new and less toxic drugs [114]. In the current study, we demonstrate a specific role for p38α signaling in cDC1s in the pathogenesis of asthma. Our results indicate that selectively targeting p38α in cDC1s is sufficient for asthma treatment. Notably, DC p38α-mediated regulation of T-cell fate during asthma development largely depends on the instructive signals encountered by the DC. Allergic or inflammatory signals acting upon DCs can induce different outcomes. Thus, we should further characterize the p38α-dependent regulation of DC function and the Th2 response, which should offer novel preventive or curative strategies for these allergic disorders.

## Supplementary information

Clean supplementary figures

Clean supplementary figures with changes in blue

Dataset S1
