# Supplementary material for: The kinase p38α functions in dendritic cells to regulate Th2-cell differentiation and allergic inflammation
Source: Cell Mol Immunol. 2022 May 12;19(7):805–19. doi: 10.1038/s41423-022-00873-2 (PMC9243149; doi:10.1038/s41423-022-00873-2)
Supplement: Supplementary file 2 — Clean supplementary figures with changes in blue [file 41423_2022_873_MOESM2_ESM.pdf]

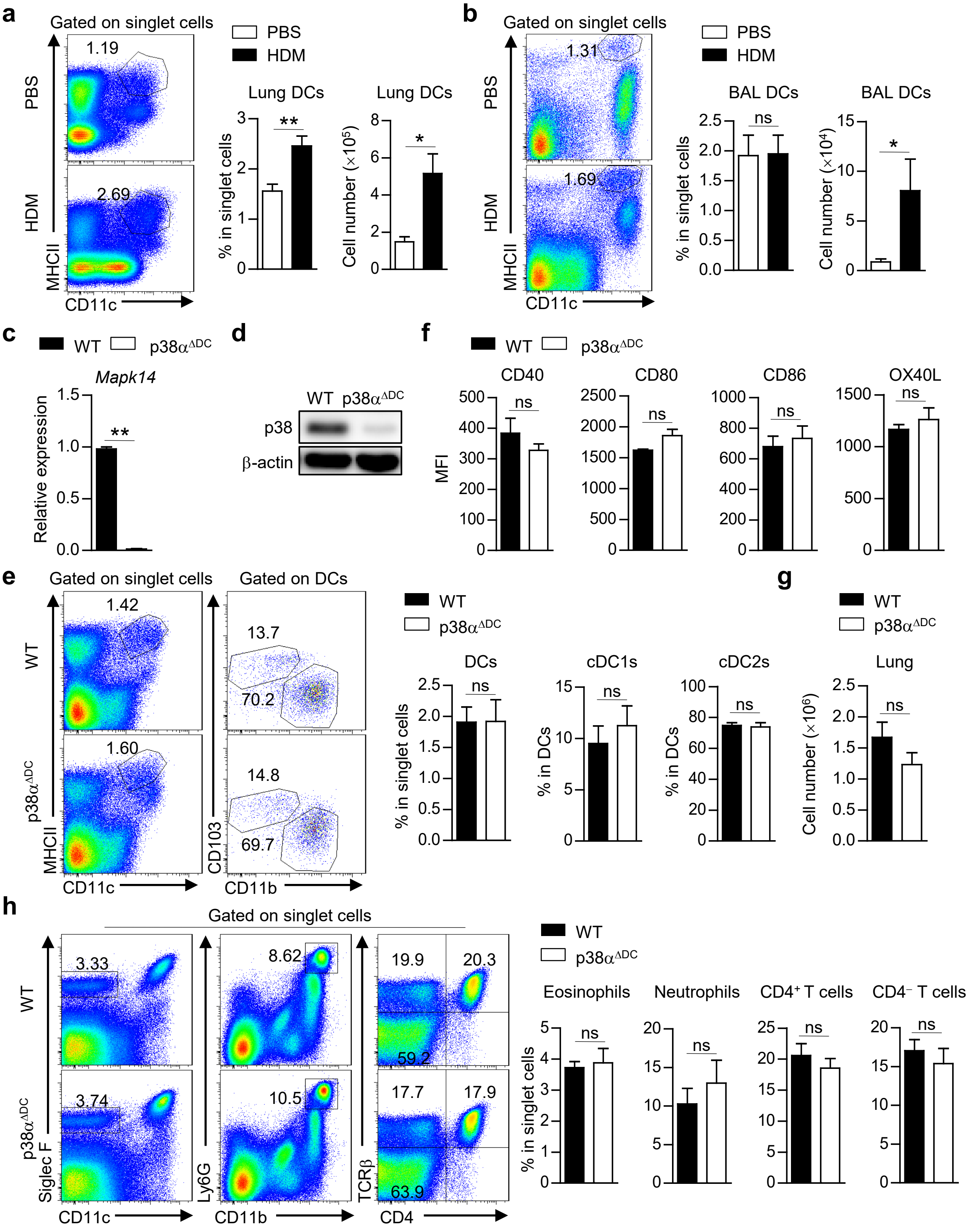

Fig. S1 HDM treatment increases DC infiltration in the lung and BAL and deletion of p38α in DCs does not affect lung immune homeostasis. (a, b) WT mice were *i.n.* sensitized with 50 μg HDM for 3 consecutive days (n = 4-5). Percentage and cell number of DCs ($CD11c^{+}MHCII^{high}$) in the lung (a)

and BAL (b) were examined 24 h after the last challenge. (c) *Mapk14* expression was examined in flow cytometry-sorted naïve WT and p38α-deficient lung DCs ($CD11c^{+}MHCII^{high}$) by qPCR (n = 3).(d) p38 expression was measured in WT and p38α-deficient splenic DCs by Western blot.(e) Flow cytometry and proportions of total DCs, cDC1 and cDC2 subsets in the lung of naïve WT and p38$\alpha^{\Delta DC}$ mice (n = 4). (f) Mean of fluorescence intensity (MFI) of CD40, CD80, CD86 and OX40L expression in naïve WT and p38α-deficient lung DCs (n = 3). (g) Total cell number isolated from lung tissues of naïve WT and p38$\alpha^{\Delta DC}$ mice (n = 3). (h) Flow cytometry (left) and proportions (right) of eosinophils, neutrophils, $CD4^{+}TCR\beta^{+}$ T cells and $CD4^{-}TCR\beta^{+}$ T cells in lung tissues of WT and p38$\alpha^{\Delta DC}$ mice (n = 3). *$P < 0.05$, **$P < 0.01$. ns, not significant. Data are representative of three (a, b and d-h) independent experiments or pooled of three independent experiments with consistent results (c). Student's *t*-test (a-c, e-h) was performed and data are presented as mean ± SEM.

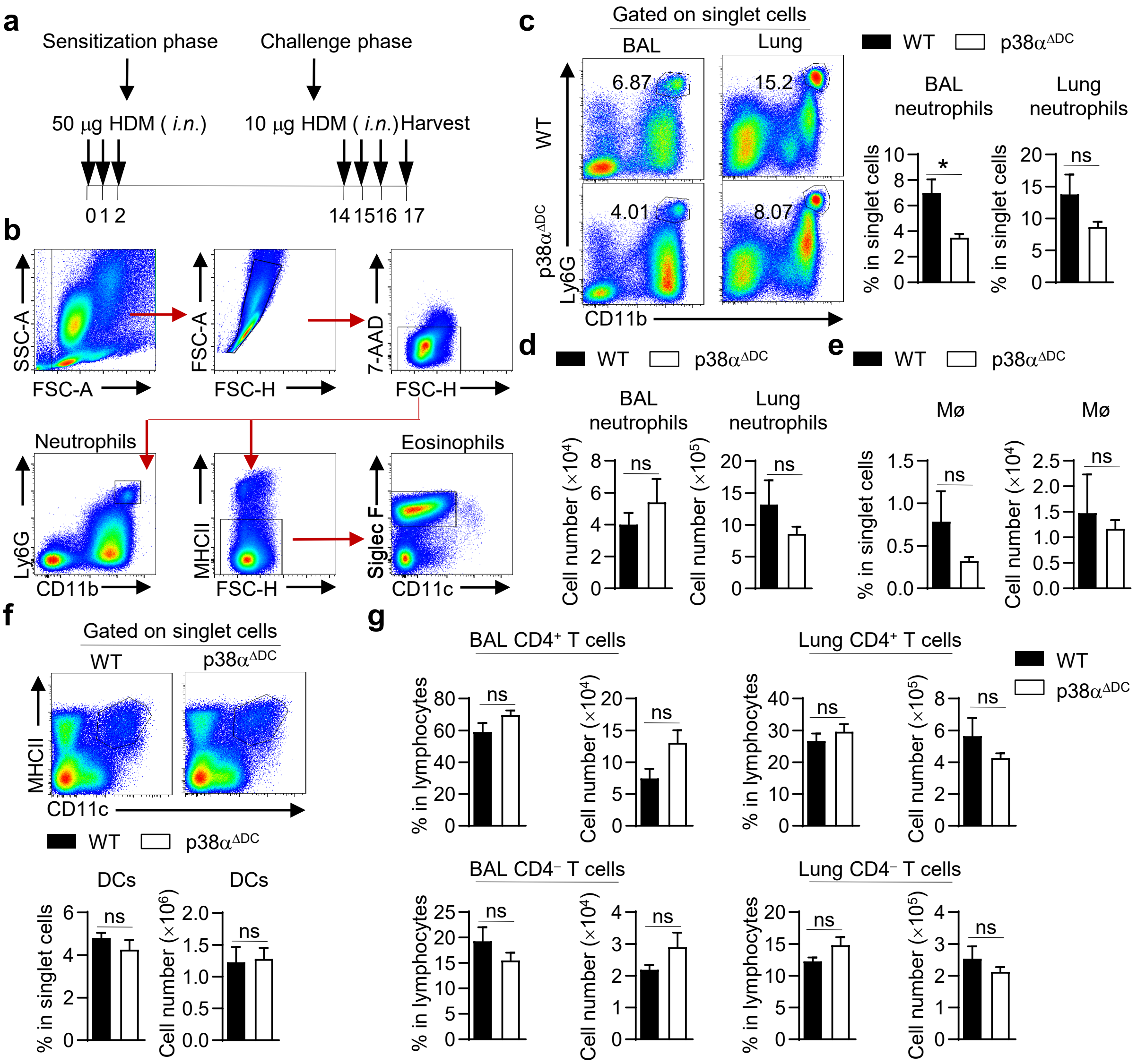

Fig. S2 Deletion of p38α in DCs does not affect cell infiltration of neutrophils, macrophages, DCs and T cells during asthma pathogenesis. (a) Experimental protocol for HDM-induced asthma. (b) Representative gating strategy for the evaluation of eosinophils and neutrophils. (c, d) Flow cytometry, proportion and cell number of neutrophils in the BAL and lungs (n = 4). (e) The frequency and cell number of macrophages (Mø) in the BAL (n = 5). (f) Flow cytometry, proportion and cell number of DCs in the lungs (n = 5). (g) The frequencies and cell numbers of

$CD4^+$ and $CD4^-$ T cells in the BAL and lungs of WT and $p38\alpha^{\Delta DC}$ mice (n = 5). *$P < 0.05$. ns, not significant. Data are representative of three (c-g) independent experiments. Student's *t*-test (c-g) was performed and data are presented as mean ± SEM.

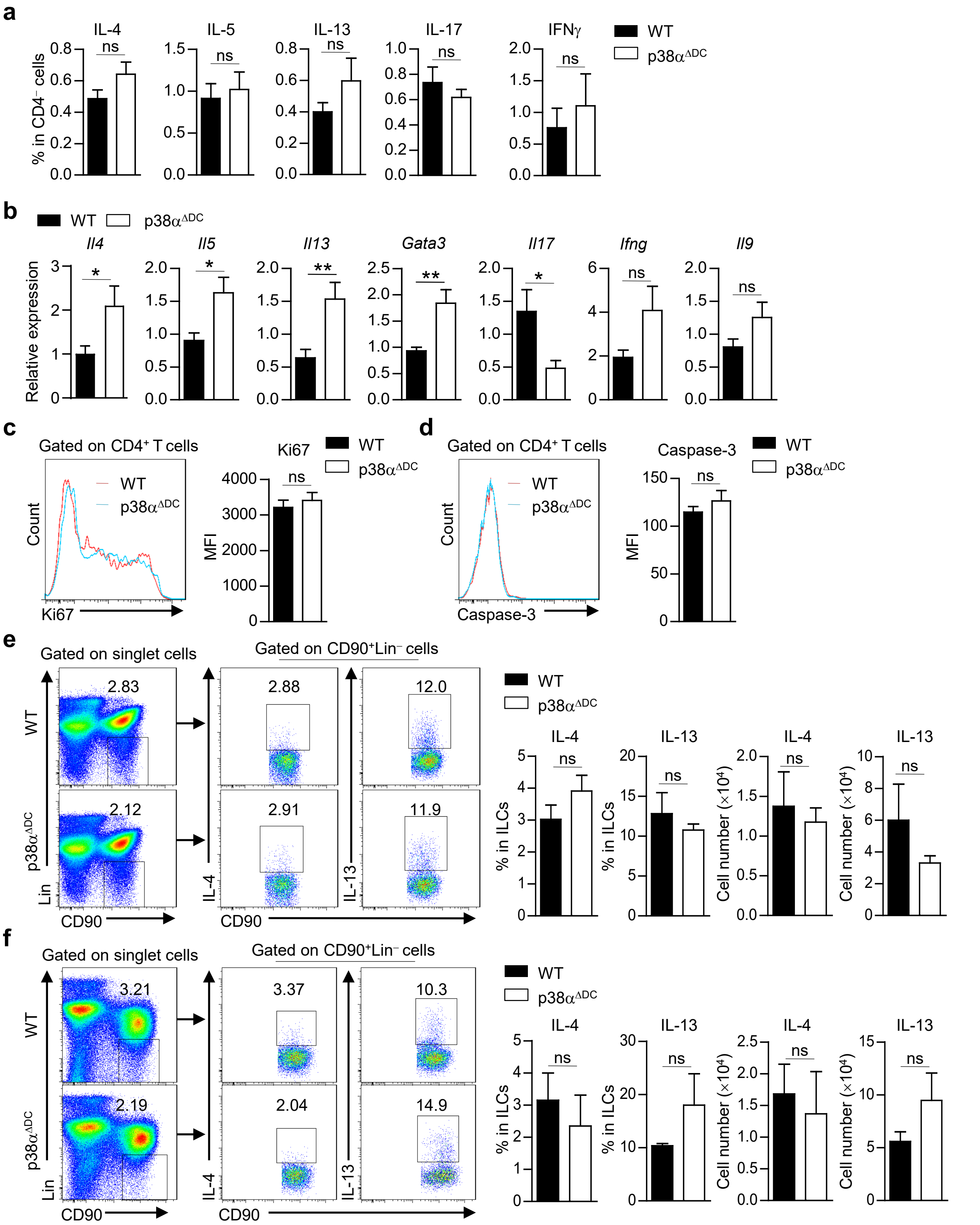

Fig. S3 p38α activity in DCs regulates Th2 responses upon HDM treatment. (a) Percentages of IL-4^{+}, IL-5^{+}, IL-13^{+}, IL-17^{+} and IFNγ^{+}CD4^{-} cells in lung tissues determined by intracellular staining (n = 13). (b) mRNA expression of T

cell-related genes in HDM-sensitized lung tissues measured by qPCR and normalized to *Hprt* (n = 9-10). (c, d) Cells were isolated from lung tissues of HDM-sensitized WT and p38α$^{\Delta DC}$ mice, and then analyzed by Ki67 and active Caspase-3 staining of $CD4^+$ T cells (n = 5). (e) Flow cytometry (left), proportions (middle) and cell numbers (right) of IL-4^+ and IL-13^+ cells in ILCs in the lung of HDM-sensitized WT and p38α$^{\Delta DC}$ mice (n = 6). (f) Flow cytometry (left), proportions (middle) and cell numbers (right) of IL-4^+ and IL-13^+ cells in ILCs in the lung of papain-sensitized WT and p38α$^{\Delta DC}$ mice (n = 3). *$P < 0.05$, **$P < 0.01$. ns, not significant. Data are pooled of three (a) or two (b) independent experiments or representative of two (c-f) independent experiments. Student's *t*-test (a-f) was performed and data are presented as mean ± SEM.

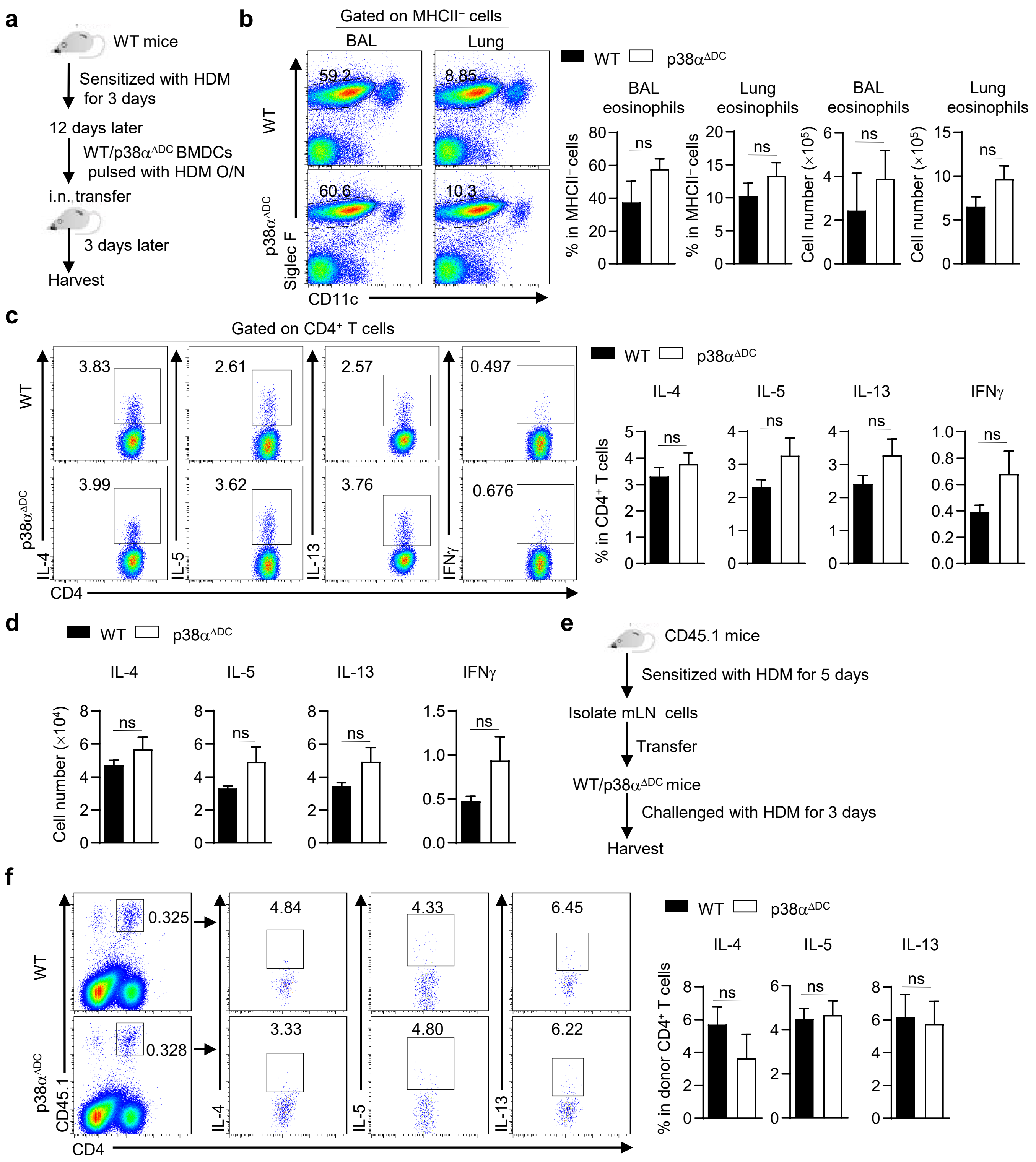

Fig. S4 p38α activity in DCs is not required for HDM-induced effector allergic inflammation. (a-d) WT mice were sensitized with HDM for 3 consecutive days. The mice were rested for 12 days and *i.n.* transferred with 2×10^6 HDM-pulsed WT or p38-deficient BMDCs, and then analyzed 72 h after BMDC transfer (n = 3-4).

Experimental schema (a). Flow cytometry, proportions and cell number of eosinophils in the BAL and Lung (b). Flow cytometry, proportions (c) and cell numbers (d) of IL-4^+, IL-5^+, IL-13^+ and IFNγ^+ CD4^+ T cells in the lung. (e and f) 10×10^6 mLN cells isolated from HDM-sensitized CD45.1 mice were *i.n.* injected into WT and p38$\alpha^{\Delta DC}$ recipient CD45.2 mice. The recipients were then *i.n.* challenged with 10 μg HDM for 3 consecutive days and analyzed 24 h after the last challenge. Experimental schema (e). Flow cytometry and proportions of donor IL-4^+, IL-5^+ and IL-13^+ CD4^+ T cells (f) (n = 3-4). ns, not significant. Data are representative of two (b-d, f) independent experiments. Student's *t*-test was performed and data are presented as mean ± SEM.

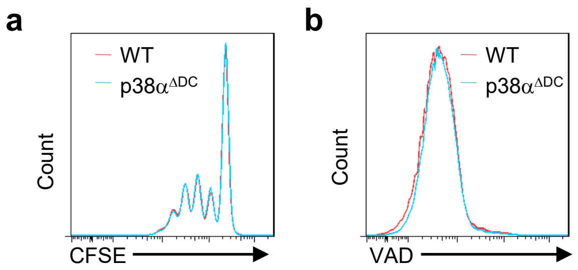

Fig S5 p38α deletion in DCs does not affect T cell proliferation or survival in vitro. Naïve OT-II $CD4^+$ T cells ($CD4^+CD25^-CD62L^+CD44^-$) co-cultured with WT and p38α-deficient lung DCs. Proliferation (a) and survival (b) of OT-II $CD4^+$ T cells were detected 72 h and 96 h later, respectively. Data are representative of two independent experiments (a, b). Every DC sample was pooled from at least three WT or p38$\alpha^{\Delta DC}$ mice.

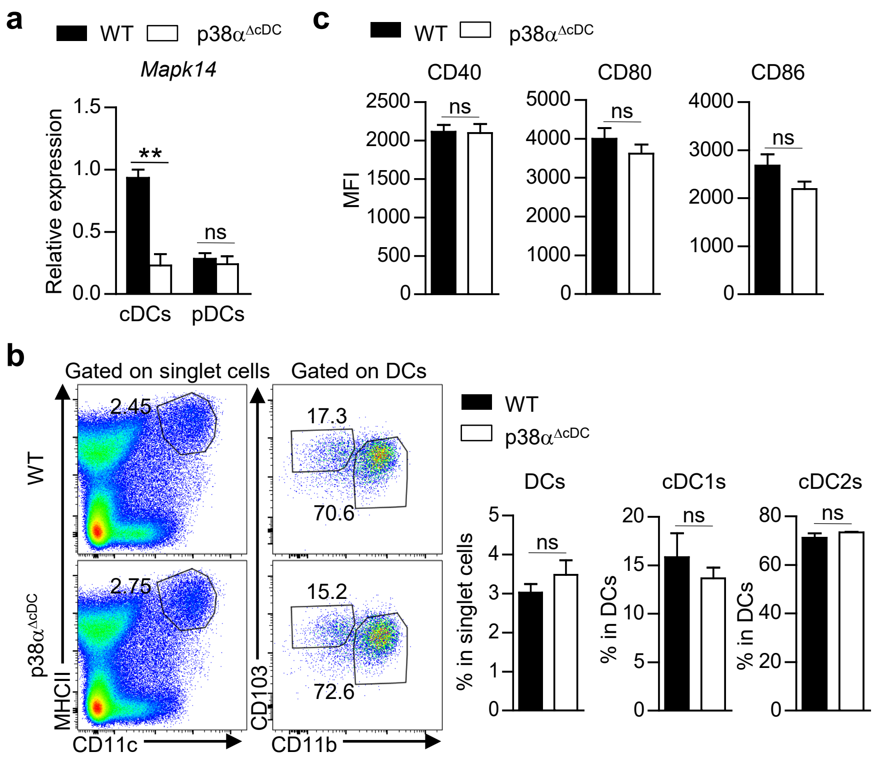

Fig. S6 p38α deletion in cDCs does not affect DC subsets and activation upon HDM treatment. (a) *Mapk14* expression in naïve WT and p38α$^{\Delta cDC}$ cDCs (CD11c^{hi}MHCII^{hi}) and pDCs (CD11c^{dim}PDCA-1^{+}) (n = 3). (b) Flow cytometry (left) and proportions (right) of total DCs, cDC1 and cDC2 subsets in the lung of HDM-treated WT and p38α$^{\Delta cDC}$ mice (n = 4). (c) MFI of CD40, CD80, CD86 expression in HDM-treated WT and p38α-deficient lung DCs (n = 4). ***P* < 0.01. ns, not significant. Data are pooled from two independent experiments (a) or representative of two independent experiments with consistent results (b, c). Two-way ANOVA (a) and student's *t*-test (b, c) was performed and data are presented as mean ± SEM.

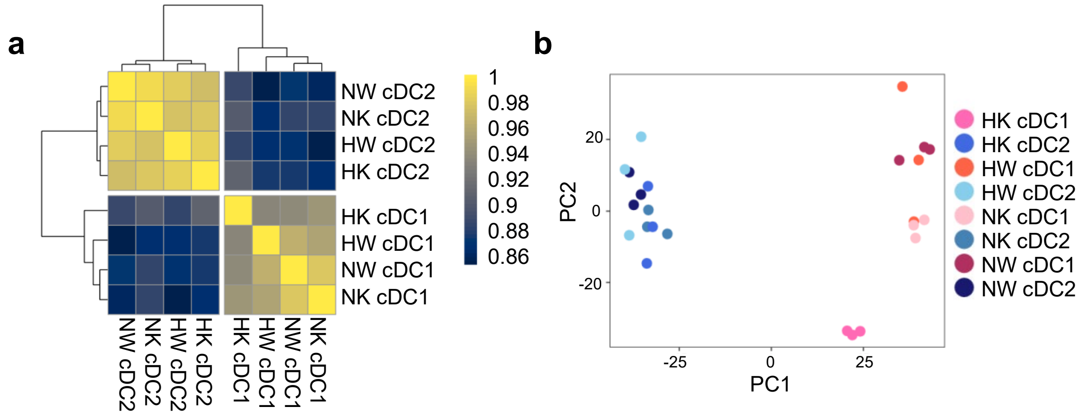

Fig. S7 The effect of p38α deficiency is more pronounced under HDM-stress in the cDC1 population. (a) Pearson correlation and clustering of cDC1s and cDC2s. (b) PCA plot of the RNAseq results. NW, NK, HW and HK are short for naïve WT, naïve p38α knock-out, HDM-treated WT and HDM-treated p38α knock-out, respectively.

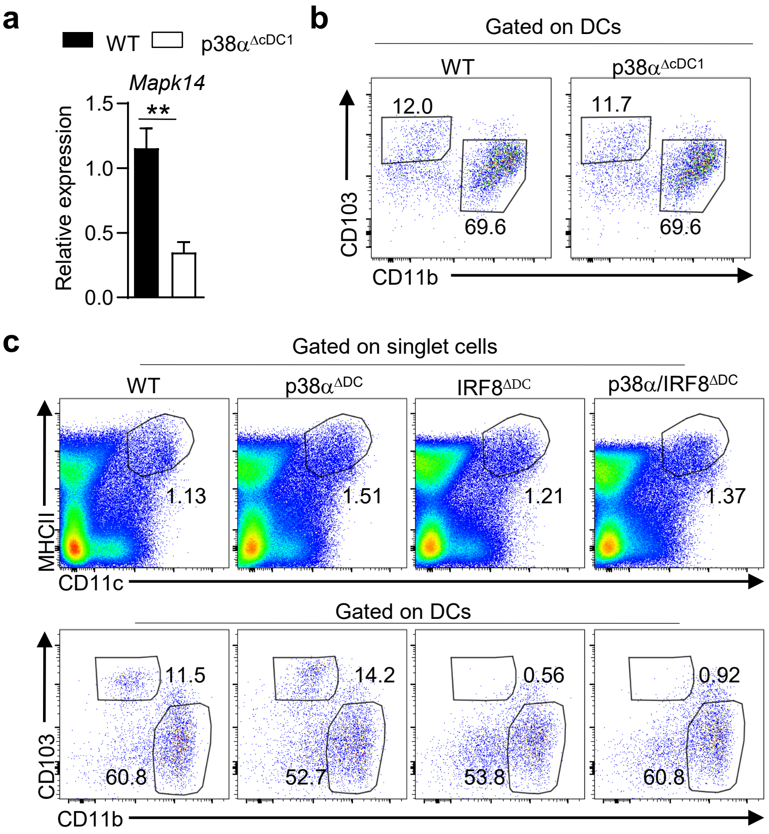

Fig. S8 p38α deletion in cDC1s does not affect DC subsets and percentage. (a) *Mapk14* expression was examined in flow cytometry-sorted naïve WT and p38α-deficient splenic cDC1s by qPCR (n = 4). (b) Flow cytometry of cDC1s and cDC2s in the lung of naïve WT and p38α$^{\Delta cDC1}$ mice. (c)The percentages of DCs, cDC1s and cDC2s were detected by flow cytometry. Data are pooled of two (a) or representative of two (b and c) independent experiments with consistent results.

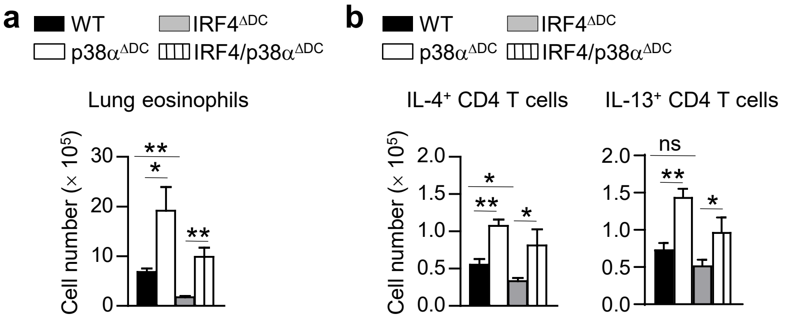

Fig. S9 p38α-deletion in DCs promotes HDM-induced lung inflammation in a cDC2-independent manner. WT, p38α$^{\Delta DC}$, IRF4$^{\Delta DC}$ and IRF4/p38α$^{\Delta DC}$ mice were sensitized and challenged with HDM for asthma induction (n = 3-5). (a) Cell number of eosinophils in lung tissues. (b) Cell numbers of IL-4^{+}CD4^{+} T cells and IL-13^{+}CD4^{+} T cells in lung tissues. *$P < 0.05$; **$P < 0.01$. ns, not significant. Data are pooled from two experiments (a, b). Student's *t*-test (a, b) was performed and data are presented as mean ± SEM.

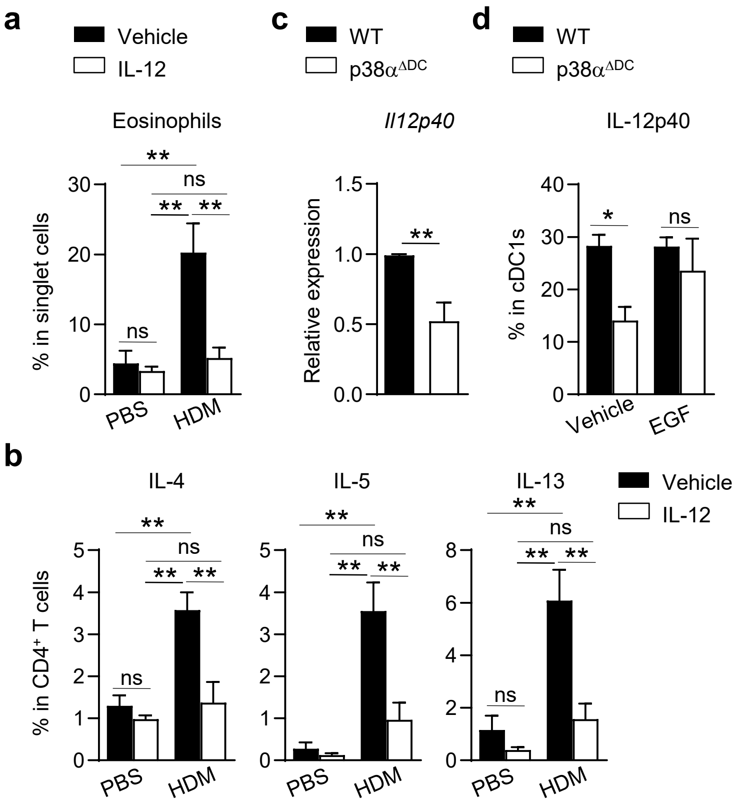

Fig. S10 p38α regulates IL-12 expression through c-FOS signaling pathway in DCs. (a and b) WT mice were induced asthma with HDM and PBS used as control. IL-12p70 was *i.n.* administrated after PBS or HDM treatment (n = 4-5). Eosinophil infiltration in lung tissues was detected by flow cytometry and quantified (a). The percentages of IL-4$^+$, IL-5$^+$ and IL-13$^+$ CD4$^+$ T cells in lung tissues were detected by flow cytometry and quantified (b). (c) mRNA expression of *Il12p40* in WT and p38-deficient Flt3L-DCs stimulated with HDM for 5 h (n = 5). (d) Percentages of IL-12p40 in HDM-stimulated WT and p38α-deficient lung cDC1s pretreated with vehicle or EGF (n = 4-5). $^{**}P < 0.01$. ns, not significant. Data are pooled of four (c) or two (d) independent experiments or representative of two (a, b) independent experiments with consistent results. Student's *t*-test was performed and data are presented as mean ± SEM.

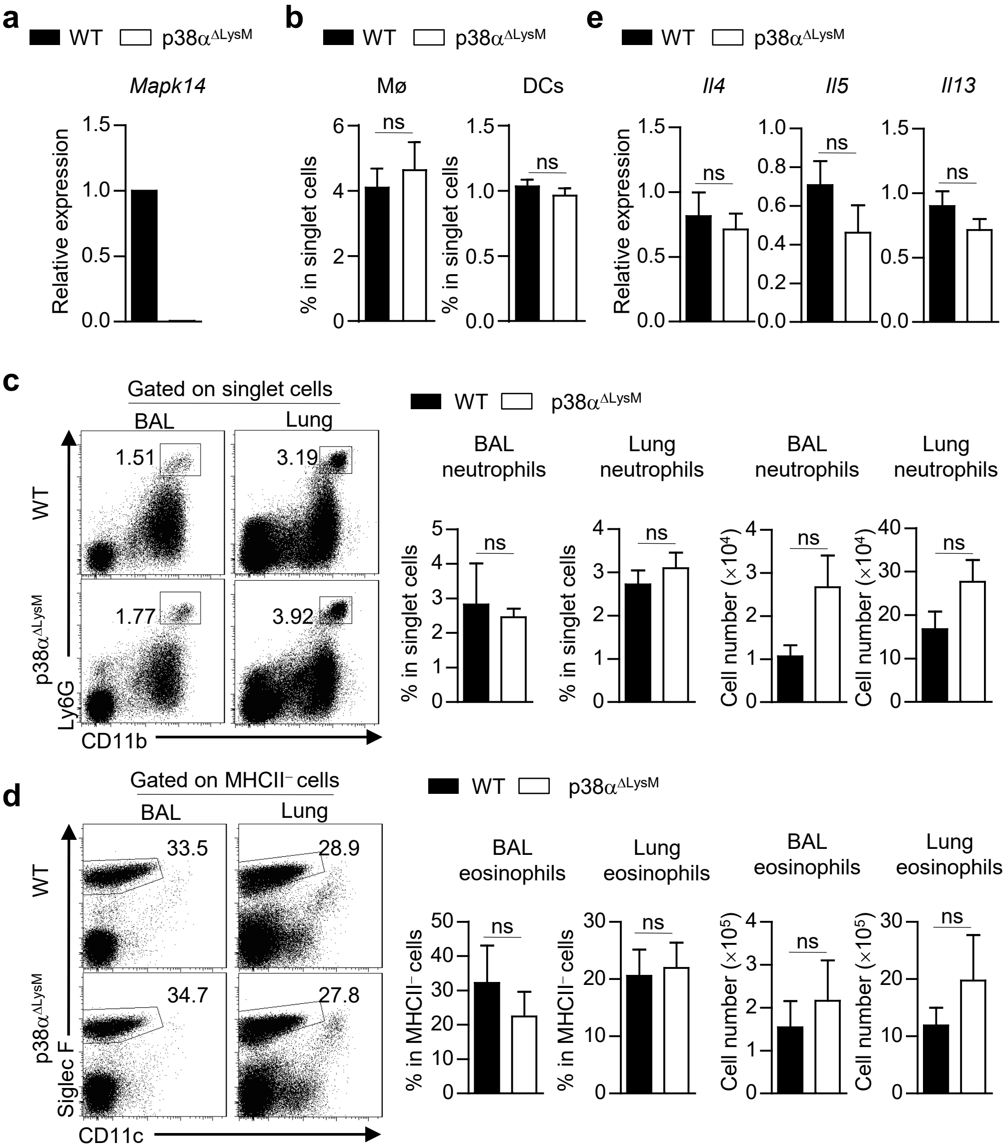

Fig. S11 p38α activity in AMs is not required for Th2-mediated allergic lung inflammation. (a) *Mapk14* expression in naïve WT and p38α$^{\Delta LysM}$ AMs (n = 1, 1 sample pooled from 3 mice). Percentages of Macrophages (Mϕ) and DCs in naïve WT and p38α$^{\Delta LysM}$ lung tissues (b) (n = 3). (c-e) WT and p38α$^{\Delta LysM}$ mice were sensitized and challenged with HDM to induce asthma. Frequencies and cell numbers of neutrophils and eosinophils in the BAL and lung tissues of HDM-treated WT and p38α$^{\Delta LysM}$ mice (c, d) (n = 4). RNA analysis of *Il4*, *Il5* and *Il13* in lung tissues of WT and p38α$^{\Delta LysM}$ mice (e) (n = 5-6). ns, not significant. Data are representative of three (a-e) independent experiments. Student's *t*-test was performed and data are presented as mean ± SEM.
